# TTK promotes mitophagy by regulating ULK1 phosphorylation and pre-mRNA splicing to inhibit mitochondrial apoptosis in bladder cancer

**DOI:** 10.1038/s41418-025-01492-w

**Published:** 2025-04-23

**Authors:** Kang Chen, Jinyu Chen, Yukun Cong, Qingliu He, Chunyu Liu, Jiawei Chen, Haoran Li, Yunjie Ju, Liang Chen, Yarong Song, Yifei Xing

**Affiliations:** 1 Department of Urology, Union Hospital, Tongji Medical College, Huazhong University of Science and Technology, Wuhan, China; 2 Department of Urology, The Second Affiliated Hospital of Fujian Medical University, Quanzhou, China

**Keywords:** Autophagy, Oncogenes, Kinases

## Abstract

Bladder cancer (BC) remains a major global health challenge, with poor prognosis and limited therapeutic options in advanced stages. TTK protein kinase (TTK), a serine/threonine kinase, has been implicated in the progression of various cancers, but its role in BC has not been fully elucidated. In this study, we show that TTK is significantly upregulated in BC tissues and cell lines, correlating with poor patient prognosis. Functional assays revealed that TTK promotes proliferation and inhibits apoptosis of BC cells. Mechanistically, TTK enhances mitophagy by directly phosphorylating ULK1 at Ser477, thereby activating the ULK1/FUNDC1-mediated mitophagy pathway. TTK knockdown disrupts mitophagy, leading to impaired clearance of damaged mitochondria, excessive accumulation of mitochondrial reactive oxygen species (mtROS), and activation of mitochondrial apoptosis. Furthermore, TTK phosphorylates SRSF3 at Ser108, preventing ULK1 exon 5 skipping and maintaining ULK1 mRNA stability. These findings show that TTK plays a key role in maintaining mitophagy in BC cells. Targeting TTK could offer a promising new approach for BC treatment by disrupting mitophagy and inducing mitochondrial apoptosis.

## Introduction

In 2023, bladder cancer (BC) was the fourth most common malignancy in men, accounting for 6% of de novo cases, and was responsible for 4% of cancer-related deaths [1, 2]. At the time of diagnosis, 70–75% of patients have non-muscle-invasive bladder cancer (NMIBC), while 20-25% have muscle-invasive bladder cancer (MIBC), and 5% develop metastatic disease. The risk of NMIBC progressing to MIBC depends on tumor grade and depth, with about 20% of patients progressing within two years [3, 4]. Despite advances in diagnosis and treatment, about 50% of MIBC patients suffered progression within five years [5]. Understanding the molecular mechanisms behind BC progression and invasion is crucial for improving treatment strategies.

The spindle assembly checkpoint (SAC) ensures proper chromosome separation during mitosis by preventing improper attachment of microtubules to kinetochores [6]. Targeting SAC proteins can induce genomic instability and apoptosis, making them promising cancer therapies [7]. Several SAC proteins, including budding uninhibited by benzimidazole (BUB), mitotic arrest deficient (MAD), and the serine/threonine kinase TTK protein kinase (TTK), are often overexpressed in cancers [6]. TTK is essential for mitotic checkpoint complex formation, chromosome alignment, cytokinesis, and DNA damage response, acting as an oncogene in cancers such as thyroid, breast, lung, prostate, and liver [7–14]. However, the exact role of TTK in BC progression remains unclear.

Mitophagy, a selective form of autophagy, removes damaged mitochondria and maintains mitochondrial integrity under stress, such as hypoxia, nutrient deprivation, and oxidative damage [15]. By removing defective mitochondria, mitophagy supports cancer cell survival and growth, contributing to tumor progression and resistance to therapy [16, 17].

Alternative splicing of pre-mRNA increases gene expression diversity and is vital for protein regulation [18]. Dysregulation of splicing factors is linked to cancer development [19, 20]. Serine/arginine-rich splicing factors (SRSFs), a class of RNA-binding proteins, play a key role in RNA splicing and modifications [21]. Phosphorylation of SR proteins stabilizes RNA binding and facilitates their nuclear-cytoplasmic shuttle [22, 23].

In this study, we found that TTK expression was upregulated in BC cells and associated with poor prognosis. Inhibiting TTK reduced ULK1 phosphorylation at Ser477, suppressed mitophagy, and promoted mitochondrial apoptosis, thus inhibiting BC progression. Additionally, TTK phosphorylated SRSF3 at Ser108, preventing ULK1 exon 5 skipping and maintaining ULK1 mRNA stability. Our research revealed that TTK plays a crucial role in activating ULK1-mediated mitophagy and regulating ULK1 pre-mRNA splicing. These findings position TTK as a promising therapeutic target in BC.

## Materials and methods

### Cell culture and reagents

Bladder cancer cell lines RT-112, UM-UC-3, TCCSUP, T24, 5637, and J82 were obtained from the Chinese Academy of Sciences Cell Bank (Shanghai, China). Cells were cultured in RPMI-1640 medium (#A4192301, Thermo Fisher Scientific, USA), supplemented with 10% fetal bovine serum (FBS), and maintained at 37 °C in a 5% CO_2_ atmosphere. The primary normal bladder epithelial cell line, BdEC (#PCS-420-010, ATCC, USA), was cultured in bladder epithelial cell basal medium supplemented with bladder epithelial growth factors.

### Clinical samples

Fresh tumor samples and adjacent non-tumor tissues from BC patients were from Wuhan Union Hospital. All patients included in the study had not received preoperative radiotherapy or chemotherapy. All procedures involving the collection of clinical samples were approved by the Ethics Committee of Union Hospital, Tongji Medical College, Huazhong University of Science and Technology, and conducted in accordance with the Declaration of Helsinki guidelines.

### Western blot analysis

Proteins were extracted from cells or tissues using RIPA buffer and quantified using a BCA Protein Assay Kit (#23227, Thermo Scientific, USA). Protein samples were separated on 8–12% SDS-PAGE gels and transferred to PVDF membranes. Membranes were blocked for 1 h and incubated overnight with primary antibodies. After incubation with species-specific secondary antibodies for 1 h, bands were detected by using an enhanced chemiluminescence system. The specific antibodies used are listed in Supplementary Table S1. Full and uncropped western blots are shown in Supplementary Material.

### Immunohistochemistry (IHC)

IHC staining was performed by using previously described methods and the TTK antibody (#10381-1-AP, Proteintech, Wuhan, China). The percentage and intensity of positively stained BC cells were combined to generate an H-score to assess immunoreactivity. Staining intensity was graded on a 4-point scale: 0 (no staining), 1 (weak staining), 2 (moderate staining), and 3 (strong staining). The H-score was calculated using the formula: (percentage of weak staining × 1) + (percentage of moderate staining × 2) + (percentage of strong staining × 3).

### Quantitative real-time PCR (qRT-PCR)

Total RNA was extracted using TRIzol reagent (#15596018CN, Invitrogen, USA). cDNA was synthesized from total RNA using the iScript cDNA Synthesis Kit (#170-8843, Bio-Rad, USA). qRT-PCR was performed on a StepOne Plus Real-Time PCR System using ChamQ SYBR qPCR Master Mix (#Q711-02, Vazyme, China), according to the manufacturer’s instructions. β-actin (ACTB) was used as an internal control. The copy number of mitochondrial DNA (mtDNA) obtained from cytosolic extracts was normalized by the copy number of nuclear DNA obtained from the whole-cell extracts. Primers were synthesized by Sangon Biotech (Shanghai, China) and are listed in Supplementary Table S2.

### Reverse transcription polymerase chain reaction (RT-PCR)

Total RNA was extracted using TRIzol reagent (#15596018CN, Invitrogen, USA) and reverse-transcribed into cDNA using the iScript cDNA Synthesis Kit (#170-8843, Bio-Rad, USA). PCR amplification was performed using specific primers covering the regions of interest. PCR products were separated on agarose gels and visualized by staining with ethidium bromide. The specific primers used are listed in Supplementary Table S2. Full and uncropped gel images are shown in Supplementary Material.

### TTK knockdown and overexpression

TTK shRNA expression vectors (pLKO.1) were constructed and transfected into RT-112 and UM-UC-3 cells, and stable knockdown clones were selected using puromycin. For TTK overexpression, Flag-tagged TTK expression vectors were constructed and transfected into cells, and stably overexpressing clones were selected using G418. Plasmid transfections were performed using ViaFect Transfection Reagent (#E4981, Promega, USA). Many of these experiments were conducted 48 hours after TTK knockdown or overexpression. For experiments involving CFSE, flow cytometry apoptosis, and MitoSOX, multiple time points (e.g., 48 h, 72 h, 96 h) were analyzed to capture dynamic changes. Relevant details are provided in the Results section. siRNA transfections were conducted using Lipofectamine RNAiMAX Reagent by following the manufacturer’s instructions. siRNAs were purchased from GenePharma, and sequences are listed in Supplementary Table S3.

### Cell proliferation, cell cycle, and apoptosis assays

Cell proliferation was measured using the CCK-8 Kit (#CK04, Dojindo Laboratories, Japan). Colony formation assays were performed by seeding cells into 6-well plates and staining colonies with crystal violet after 14 days of culture. EdU incorporation assays were conducted using the Click-iT EdU Imaging Kit (#C10353, Invitrogen, USA). CFSE staining was used to assess cell proliferation by monitoring the dilution of CFSE fluorescence over time in RT-112 and UM-UC-3 cells. To improve the accuracy of cell cycle analysis, BC cells were synchronized in the G1 phase using thymidine treatment before TTK knockdown. After 48 h, both floating and adherent cells were collected for cell cycle and apoptosis analysis. Cell cycle distribution and apoptosis rates were flow cytometrically analyzed by using propidium iodide (PI) staining and FITC Annexin V and PI staining were used for apoptosis detection.

### In vivo tumor growth assays

Four-week-old male athymic BALB/c nude mice were bought from Beijing Vital River Laboratory Animal Technology Co., Ltd. RT-112 cells (1 × 10^6^) were subcutaneously injected into the flank of each mouse. For in vivo administration of the small molecule inhibitor CFI-402257 (#1610759-22-2, MCE, China), mice were orally gavaged at 6 mg/kg/day of CFI-402257 or vehicle (90% PEG in ultrapure water) on daily basis, starting from day 14 post-injection. Tumor volumes were measured and recorded using the formula: Volume = 0.5 × Length × Width^2^. At the end of the experiment, tumors were harvested, weighed, and then analyzed.

### Transmission electron microscopy

Cells were collected [24], pelleted, and fixed in 2.5% glutaraldehyde at 4 °C for 2 h. Transmission electron microscopy imaging was performed by Servicebio (Wuhan, China).

### Mt-Keima mitophagy detection

To assess mitophagy, RT-112 and UM-UC-3 cells were infected with mt-Keima-expressing adenovirus (HANBIO, Shanghai, China) at an MOI of 50, followed by treatments. After washing with PBS, cells were observed under a confocal microscope. Mt-Keima fluorescence was measured using 440 nm (neutral pH) and 550 nm (acidic pH) excitation wavelengths, with emission collected at 620 nm. The fluorescence intensity ratio (550 nm/440 nm) was calculated, where a higher ratio indicated increased mitophagy. Fluorescence at 440 nm excitation was pseudo-colored green, and at 550 nm excitation, red.

### Plasmid transfection

GFP-LC3B (#P36235) expression plasmids were provided by Invitrogen, and HBLV-Cox8-EGFP-mCherry plasmids were purchased from HANBIO. According to the manufacturer’s instructions, cells were transfected using Lipofectamine 3000 (#L3000015, Invitrogen, USA). For GFP-LC3B transfection, RT-112 and UM-UC-3 cells were transfected to stably express GFP-LC3B. After transfection, GFP-LC3B-expressing cells were labeled with MitoTracker Red (25 nM, #M7512, Invitrogen, USA) to observe mitochondrial morphology. Changes in GFP-LC3B puncta were then examined using a confocal microscope. For HBLV-Cox8-EGFP-mCherry transfection, cells were transfected and subsequently treated. The number of mCherry-positive dots, representing mitolysosomes, was quantified using confocal microscopy to assess mitophagy.

### Reactive oxygen species (ROS) detection

ROS levels were assessed using multiple methods. MitoSOX Red (#M36007, Invitrogen, USA) was used to specifically measure mitochondrial ROS (mtROS) production. MitoP/MitoB assays were performed to detect mitochondrial hydrogen peroxide (H_2_O_2_) levels. Both MitoB and MitoP are quantified by high-performance liquid chromatography-tandem mass spectrometer (LC–MS/MS). For general intracellular ROS detection, DHE staining (#104821-25-2, MCE, China) and DCFH-DA (#S0035S, Beyotime, China) flow cytometry were used. Fluorescence intensities from each assay were quantified to evaluate the ROS levels in RT-112 and UM-UC-3 cells.

### Mitochondrial membrane potential assays

Mitochondrial membrane potential was assessed using two different methods. JC-1 staining (#C2006, Beyotime, China) was employed to measure mitochondrial membrane potential by flow cytometry, with a shift in fluorescence indicating disruption of the membrane potential. Additionally, mitochondrial membrane potential was quantified using TMRE (Tetramethylrhodamine, ethyl ester) staining (#T669, Invitrogen, USA), where fluorescence intensity was measured to evaluate changes in mitochondrial membrane integrity in RT-112 and UM-UC-3 cells.

### Immunofluorescence staining and confocal microscopy

BC cells grown on confocal dishes were fixed with 4% paraformaldehyde for 30 min and permeabilized with 0.1% Triton X-100 for 10 minutes. After blocking with 1% BSA for 1 h, cells were incubated with primary antibodies overnight at 4 °C, followed by washing with PBST and incubation with secondary antibodies for 1 h. Details of the primary antibodies are provided in Supplementary Table S1. Samples were mounted with a DAPI-containing medium. For mitophagy and mitochondrial analysis, cells were stained with MitoTracker Red (25 nM, #M7512, Invitrogen, USA), MitoTracker Green (50 nM, #M7514, Invitrogen, USA), and LysoTracker Red (50 nM, #L7528, Invitrogen, USA). Imaging was performed using a Nikon A1Si confocal microscope.

### Recombinant protein expression and purification

Recombinant TTK and ULK1 proteins, tagged with GST, were purchased from Sino Biological. SRSF3, tagged with His, was expressed using the E. coli expression system and purified in-house using Beyotime’s His-tag Protein Purification Kit (Reductive Chelating Resin). The purified recombinant proteins were used for subsequent in vitro kinase assays and mass spectrometry (MS) analysis. Details of the recombinant TTK and ULK1 proteins used are provided in Supplementary Table S1.

### RNA sequencing (RNA-Seq) and splicing analysis

By following the manufacturer’s instructions, total RNA extracted from RT-112 cells transfected with negative control (shNC) and shTTK was subjected to 150 bp paired-end RNA-Seq on an Illumina NovaSeq platform. Differential alternative splicing events were analyzed by using rMATS (version 3.2.5) software, which categorizes alternative splicing events into five types: exon skipping, intron retention, alternative 5′ splice site, alternative 3′ splice site, and mutually exclusive exons. The software performs differential alternative splicing analysis on samples with biological replicates. Each alternative splicing event corresponds to two isoforms: exon inclusion and exon skipping. The expression levels of the isoforms were normalized by their effective length, and the proportion of the exon inclusion isoform (IncLevel) was calculated. The significance of differential expression was then assessed. Exon skipping events were validated by RT-PCR, and PCR products were analyzed by electrophoresis. The specific primers for each mRNA are listed in Supplementary Table S2.

### Minigene construction

Minigenes were constructed as previously described [25]. Briefly, the GFP-ULK1-WT minigene was created by cloning a sequence containing exons 1–6 along with 300 bp of the flanking intronic regions into the pcDNA3.1(+) vector. By using this GFP-ULK1-WT plasmid as a template, site-directed mutations were introduced to mutate the GCAACGG sequence in exon 5 to ACGCCTT, resulting in the GFP-ULK1-Mut minigene. The constructed minigenes were verified by DNA sequencing.

### Immunoprecipitation–mass spectrometry (IP-MS) analysis

Flag-TTK expression vectors were transfected into RT-112 cells. Immunoprecipitation was performed using Flag antibodies, and co-immunoprecipitated proteins were visualized by silver staining and identified by MS. Interacting proteins were validated by co-immunoprecipitation (co-IP), and SRSF3 binding sites were predicted by using beRBP [26].

### In vitro kinase assays

Recombinant TTK kinase and recombinant ULK1/SRSF3 proteins were incubated in a kinase reaction buffer containing ATP. Phosphorylation levels were detected by Western blot, and phosphorylation sites were mass spectrometry identified.

### RNA immunoprecipitation (RIP) assays

RIP assays were performed to analyze the interaction between SRSF3 and ULK1 pre-mRNA. Cells were lysed and incubated with antibodies against SRSF3 or control IgG, followed by precipitation using protein A/G agarose beads. RNA was extracted from the immunoprecipitates and analyzed by qRT-PCR to determine the binding of SRSF3 to ULK1 pre-mRNA.

### Data analysis

Data analysis was performed using GraphPad Prism 9 statistical software. Data are presented as the mean ± standard deviation (SD). For comparisons between two groups, two-tailed paired or unpaired Student’s t-tests were applied as appropriate. For multiple group comparisons, one-way or two-way analysis of variance (ANOVA) was used. Survival differences were analyzed by log-rank test (Kaplan–Meier survival curves). Pearson correlation assessed mRNA expression relationships in the TCGA BLCA dataset. Statistical significance was defined as *P* < 0.05. Significance levels are denoted as follows: ns, not significant; *P < 0.05; **P < 0.01; ***P < 0.001.

## Results

### TTK is overexpressed in bladder cancer and correlated with poor prognosis

**Fig. 1:**
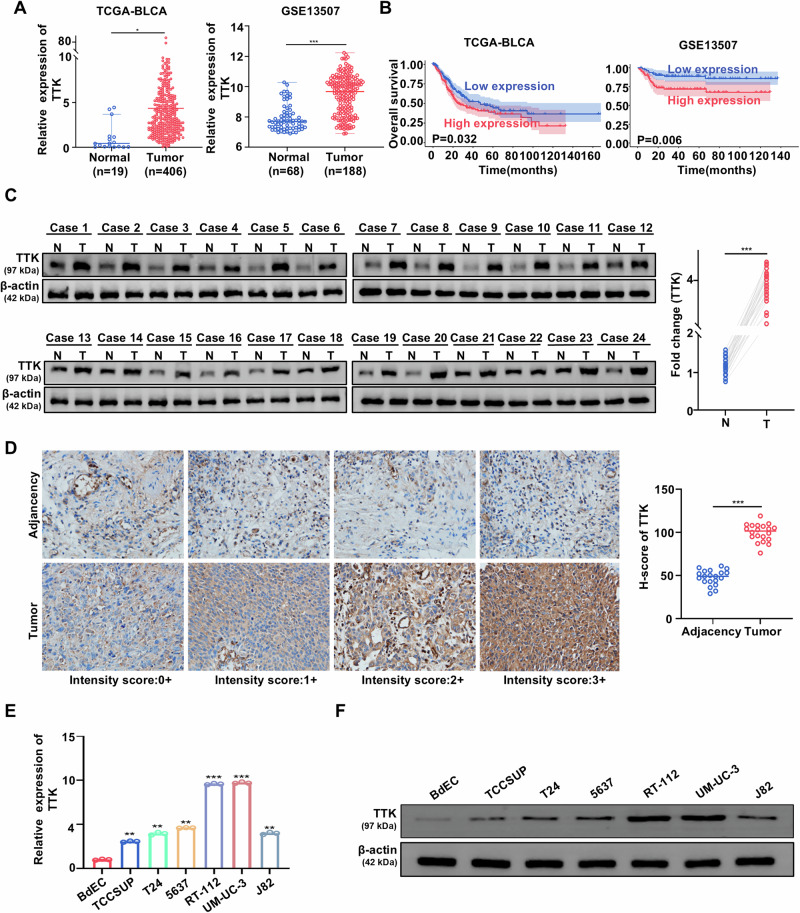
TTK is upregulated in BC and associated with poor prognosis. **A** Relative expression of TTK in normal bladder tissues and tumor tissues in the TCGA BLCA and GSE13507 datasets. **B** Kaplan–Meier survival curves showing the overall survival of patients with low and high TTK expression from the TCGA BLCA and GSE13507 datasets. **C** Western blot analysis of TTK expression in BC tissues and adjacent normal tissues (n = 24). Densitometric and statistical analysis. **D** Immunohistochemical staining showing TTK expression levels in BC tissues and corresponding adjacent tissues (original magnification, ×400), quantitatively rated using histochemical scoring (n = 20). (E and F) TTK mRNA and protein expression levels in BC cell lines (TCCSUP, T24, 5637, RT-112, UM-UC-3, and J82) and the primary bladder epithelial cell line BdEC as detected by qRT-PCR (**E**) and Western blot (**F**). Data are expressed as mean ± SD from three independent experiments. *P < 0.05; **P < 0.01; ***P < 0.001.

To assess the implication of TTK expression in BC progression, we performed differential expression analysis using the TCGA BLCA and GSE13507 datasets. Compared to normal tissues, TTK was significantly upregulated in BC tissues (Fig. 1A). We then evaluated the prognostic value of TTK in the TCGA BLCA and GSE13507 datasets, finding that high TTK expression was significantly associated with poor prognosis (Fig. 1B). We further examined TTK protein expression in BC tissues. As expected, TTK protein levels were significantly higher in BC tissues compared to adjacent normal tissues (Fig. 1C, D). Additionally, TTK was overexpressed in various types of solid tumors, underscoring its significance in cancer progression (Supplementary Fig. S1A–R). Furthermore, we observed that all BC cell lines (TCCSUP, T24, 5637, RT-112, UM-UC-3, and J82) exhibited higher TTK mRNA and protein levels compared to the primary bladder epithelial cell line BdEC (Fig. 1E, F). Overall, these results suggest that elevated TTK expression might indicate an unfavorable prognosis in BC patients.

### TTK promotes proliferation and inhibits apoptosis of BC cells

**Fig. 2:**
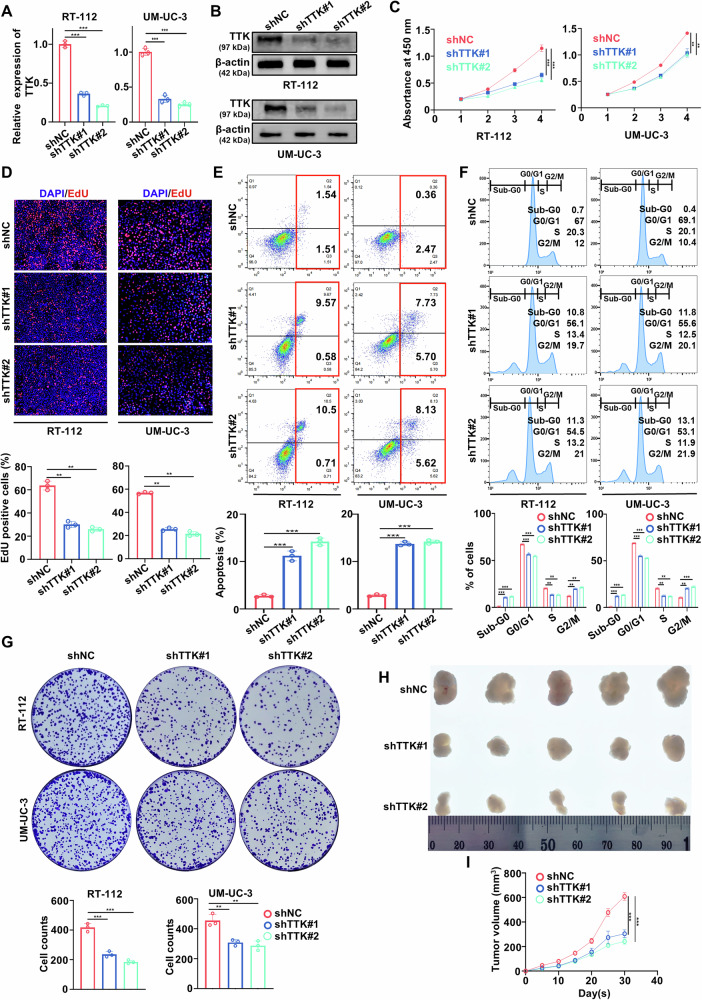
TTK knockdown inhibits proliferation of BC cells in vitro and in vivo. Knockdown efficiency of TTK in RT-112 and UM-UC-3 cells as determined by qRT-PCR (**A**) and Western blot (**B**). **C** CCK-8 assays showing cell viability of RT-112 and UM-UC-3 cells stably transfected with negative control (shNC), shTTK#1, or shTTK#2. **D** EdU assays exhibiting proliferation of RT-112 and UM-UC-3 cells stably transfected with shNC, shTTK#1, or shTTK#2. (E and F) Flow cytometry showing apoptosis (**E**) and cell cycle distribution (**F**) of RT-112 and UM-UC-3 cells stably transfected with shNC, shTTK#1, or shTTK#2 at 48 h. **G** Colony formation assays of RT-112 and UM-UC-3 cells stably transfected with shNC, shTTK#1 or shTTK#2. (H and I) Xenograft tumors (**H)** generated by subcutaneous injection of RT-112 cells stably transfected with shNC, shTTK#1, or shTTK#2 into nude mice and in vivo growth curves (**I**). Data are given as mean ± SD from three independent experiments. **P < 0.01; ***P < 0.001.

To further investigate the impact of TTK on BC growth, we employed RT-112 and UM-UC-3 cells, which highly express TTK, for subsequent studies. We first constructed stable TTK-knockdown RT-112 and UM-UC-3 cell lines (Fig. 2A, B). CCK-8 assays, CFSE staining, and EdU (5-ethynyl-2’-deoxyuridine) assays all demonstrated that TTK knockdown significantly inhibited the proliferation of RT-112 and UM-UC-3 cells (Fig. 2C, D and Supplementary Fig. S2A).

Flow cytometry analysis revealed that TTK knockdown induced significant apoptosis in both RT-112 and UM-UC-3 cells, and this effect increased over time (Fig. 2E and Supplementary Fig. S2B). Additionally, flow cytometric studies indicated that TTK knockdown significantly increased the proportion of cells in the Sub-G0 and G2/M phase (Fig. 2F), consistent with its role in promoting apoptosis and cell cycle arrest.

To evaluate the long-term effects of TTK modulation, colony formation assays and in vivo tumor xenograft studies were conducted. Colony formation assays revealed a marked reduction in cell survival, reflecting the combined impact of apoptosis promotion and cell cycle arrest (Fig. 2G). Similarly, in vivo xenograft tumors with TTK knockdown grew more slowly than their counterparts without TTK knockdown, further supporting the role of TTK in tumor progression (Fig. 2H, I).

To further assess the potential therapeutic role of TTK inhibition, we treated tumor-bearing mice with the TTK inhibitor CFI-402257. As shown in Supplementary Fig. S2C, representative images of xenograft tumors demonstrated that CFI-402257 treatment significantly reduced tumor size compared to the control group. Supplementary Fig. S2D illustrates the survival rates of tumor-bearing mice, where CFI-402257 treatment significantly prolonged survival, further supporting the potential of TTK inhibition as a therapeutic strategy for BC. Collectively, our results confirm that TTK functions as an oncogene, promoting BC cell proliferation and inhibiting apoptosis. Importantly, the therapeutic potential of targeting TTK using CFI-402257 demonstrates promise as a novel treatment approach for BC.

### TTK enhances mitophagy in BC cells

**Fig. 3:**
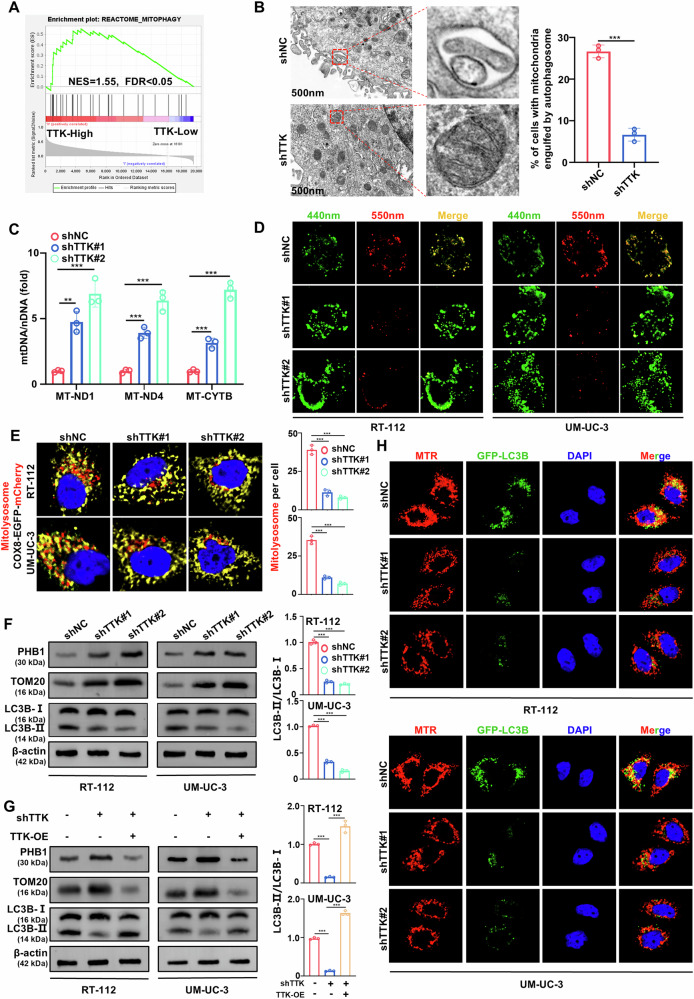
TTK knockdown inhibits mitophagy. **A** Gene Set Enrichment Analysis (GSEA) showing the association between TTK and mitophagy. An FDR < 25% was considered statistically significant. **B** Representative transmission electron microscopy images exhibiting the ultrastructure of RT-112 cells stably transfected with shNC or shTTK. (Scale bar: 500 nm). **C** Quantification of relative mtDNA copy number (MT-ND1, MT-ND4, and MT-CYTB) in RT-112 cells stably transfected with shNC, shTTK#1, or shTTK#2, as measured by qRT-PCR. **D** Representative confocal images are of RT-112 and UM-UC-3 cells expressing mt-Keima transfected with shNC, shTTK#1, or shTTK#2. (Scale bar: 10 μm). **E** Representative confocal images of RT-112 and UM-UC-3 cells transfected with the COX8-EGFP-mCherry plasmid. Quantification of mCherry dots (mitolysosomes) in indicated conditions (Scale bar: 5 μm). **F** Western blot analysis of mitophagy-related proteins in RT-112 and UM-UC-3 cells transfected with shNC, shTTK#1, or shTTK#2. Quantification of LC3B-II/LC3B-I ratio. **G** Western blot analysis of mitophagy-related proteins in RT-112 and UM-UC-3 cells after TTK overexpression rescue following TTK knockdown, and quantification of the LC3B-II/LC3B-I ratio. **H** Confocal microscopy was conducted to detect the spatial colocalization of MitoTracker Red (MTR) and GFP-LC3B in RT-112 and UM-UC-3 cells stably transfected with shNC, shTTK#1, or shTTK#2. (Scale bar: 10 μm). Data are presented as mean ± SD from three independent experiments. **P < 0.01; ***P < 0.001.

To explore the mechanisms by which TTK knockdown inhibits BC cells growth and induces apoptosis, we conducted gene set enrichment analysis (GSEA) using the TCGA BLCA dataset and found that TTK expression was positively correlated with the mitophagy level (Fig. 3A). Transmission electron microscopy showed the presence of mitochondria undergoing single-membrane engulfment in control BC cells. In contrast, the number of mitochondria located within autophagosomes or autolysosomes was significantly reduced in TTK knockdown cells (Fig. 3B). When mitochondrial function is impaired, such as through loss of membrane potential or increased oxidative stress, cells selectively remove these damaged mitochondria via mitophagy to maintain mitochondrial quality. During this process, mtDNA in damaged mitochondria is also degraded in this process [27]. Our experiments showed that mtDNA levels were significantly elevated after TTK knockdown, suggesting impaired mitophagy and the accumulation of dysfunctional mitochondria (Fig. 3C).

To further confirm the relationship between TTK and mitophagy, we examined the effect of TTK knockdown using the mt-Keima reporter gene. In RT-112 and UM-UC-3 cells, TTK knockdown led to a significant reduction in fluorescence excited at 550 nm, while fluorescence excited at 440 nm was increased, indicating impaired mitophagy (Fig. 3D and Supplementary Fig. S3A). To further validate this, we used the COX8-EGFP-mCherry plasmid, which targets mitochondria and is commonly used to monitor mitophagy [28]. Normally, mitochondria display yellow fluorescence, but those engulfed by lysosomes show only red fluorescence (Supplementary Fig. S3B). After TTK knockdown, we observed a significant decrease in the mCherry signal representing mitochondrial lysosomes (mitolysosomes) (Fig. 3E). To further validate these findings, we performed a co-localization analysis using LysoTracker Red and MitoTracker Green dyes [29]. The results showed that TTK knockdown significantly reduced mitochondrial-lysosome co-localization (Supplementary Fig. S3C). Additionally, TTK knockdown resulted in increased expression of the outer mitochondrial membrane protein TOM20 and the inner mitochondrial membrane protein PHB1, along with a decrease in the autophagy marker LC3B-II, suggesting an accumulation of mitochondrial proteins (Fig. 3F). Conversely, in rescue experiments, re-expression of TTK reduced TOM20 and PHB1 levels and increased LC3B-II expression (Fig. 3G). Moreover, TTK knockdown significantly decreased the accumulation of LC3B puncta and reduced mitochondrial-GFP-LC3B co-localization (Fig. 3H and Supplementary Fig. S3D). These findings indicate that TTK enhances mitophagy in BC cells.

### TTK inhibits mitochondrial apoptosis in BC cells by promoting mitophagy

When mitophagy is inhibited, damaged mitochondria cannot be cleared in a timely manner, leading to their accumulation. This mitochondrial dysfunction results in the release of large amounts of mtROS, which further damage other mitochondria and organelles, disrupt cellular homeostasis, and create a vicious cycle of exacerbating mitochondrial dysfunction, ultimately leading to apoptosis [30, 31].

**Fig. 4:**
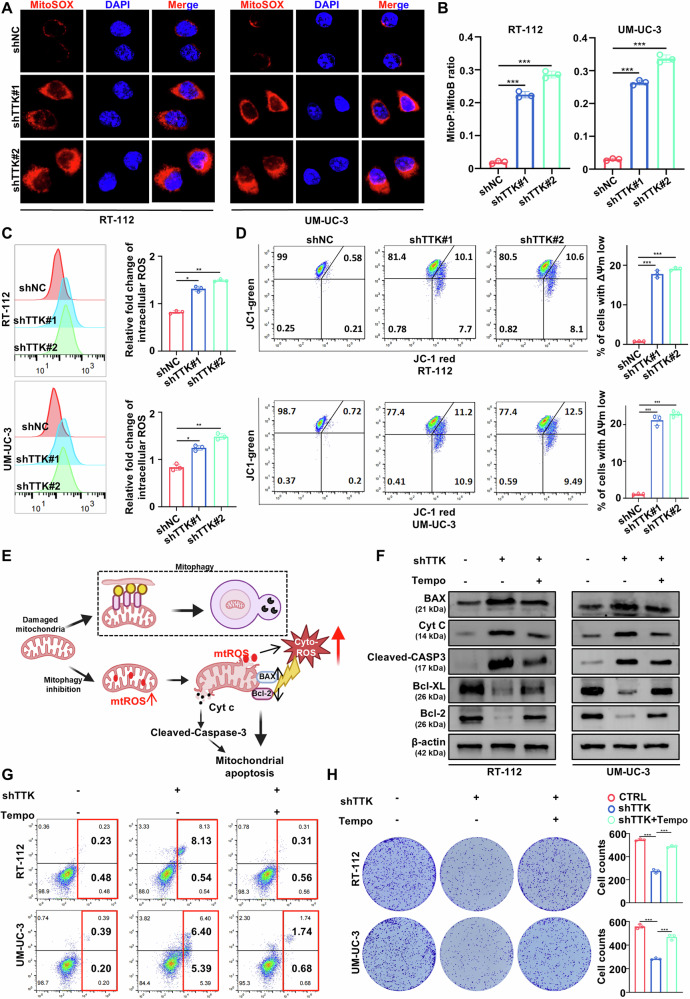
TTK knockdown promotes mitochondrial apoptosis of BC cells. **A** Representative immunofluorescence images of MitoSOX staining and quantification of MitoSOX mean fluorescence intensity (MFI) in RT-112 and UM-UC-3 cells stably transfected with shNC, shTTK#1, or shTTK#2 at 48 h. (Scale bar: 10 μm). **B** Mitochondrial H_2_O_2_ production in RT-112 and UM-UC-3 cells stably transfected with shNC, shTTK#1, or shTTK#2, assessed by MitoB oxidation. **C** Intracellular reactive oxygen species (ROS) levels in RT-112 and UM-UC-3 cells transfected with shNC, shTTK#1, or shTTK#2, were assessed by DCFH-DA staining and flow cytometry. **D** Mitochondrial membrane potential in RT-112 and UM-UC-3 cells transfected with shNC, shTTK#1, or shTTK#2 was assessed by JC-1 staining and flow cytometry. **E** Schematic diagram illustrating the proposed mechanism by which mitophagy inhibition leads to accumulation of damaged mitochondria, excessive mitochondrial ROS (mtROS) production, and induction of mitochondrial apoptosis. **F** Western blot analysis of apoptosis-related proteins in TTK knockdown RT-112 and UM-UC-3 cells, with additional evaluation after treatment with the mtROS scavenger Tempo. **G** Flow cytometry analysis of apoptosis in TTK knockdown RT-112 and UM-UC-3 cells, with additional evaluation after treatment with the mtROS scavenger Tempo. **H** Colony formation assays of TTK knockdown RT-112 and UM-UC-3 cells, with additional evaluation after treatment with the mtROS scavenger Tempo. Data are expressed as mean ± SD from three independent experiments. *P < 0.05; **P < 0.01; ***P < 0.001.

To investigate the role of TTK knockdown in mtROS production, we conducted several assays. MitoSOX staining revealed that TTK knockdown significantly increased mtROS levels over time, suggesting that TTK knockdown promotes apoptosis in BC cells by inhibiting mitophagy and accumulating mtROS (Fig. 4A and Supplementary Fig. S4A). To further confirm these findings, we used the mitochondrial-targeted H_2_O_2_ probe MitoB and observed a significant increase in the MitoP/MitoB ratio following TTK knockdown, indicating elevated mitochondrial H_2_O_2_ levels (Fig. 4B). Additionally, increased ROS generation was confirmed by higher DHE fluorescence intensity and flow cytometry analysis, which demonstrated that TTK knockdown elevated overall ROS levels in BC cells (Fig. 4C and Supplementary Fig. S4B). Mitochondrial membrane potential was determined by flow cytometry using JC-1 staining and further confirmed by measuring fluorescence intensity using the TMRE dye (Fig. 4D and Supplementary Fig. S4C). These changes were accompanied by a loss of mitochondrial membrane potential, further supporting the role of TTK knockdown in disrupting mitochondrial function.

Under normal physiological conditions, the mitochondrial inner membrane maintains an electrochemical gradient essential for energy production and respiration. However, when mtROS production increases due to stress or damage, this electrochemical gradient dissipates, resulting in mitochondrial dysfunction. A key mechanism by which mtROS contributes to apoptosis is through interactions with mitochondrial proteins, leading to oxidative modifications that trigger the opening of the mitochondrial permeability transition pore, causing a loss of mitochondrial membrane potential and the release of pro-apoptotic factors, such as cytochrome c (Cyt C), into the cytosol. Once in the cytosol, Cyt C binds to Apaf-1, forming the apoptosome, which subsequently activates caspase-9. Activated caspase-9 then cleaves and activates caspase-3, the executioner caspase, leading to the cleavage of various cellular substrates and ultimately resulting in apoptosis. Additionally, mtROS may also leak from the inner membrane into the cytosol, further increasing cytosolic ROS levels. When mitophagy is inhibited, dysfunctional mitochondria accumulate, resulting in elevated cytosolic levels of Cyt C and ROS. These molecules activate caspase-dependent apoptotic pathways and pro-apoptotic proteins of the Bcl-2 family, such as Bax. Additionally, ROS activates Bad through JNK phosphorylation, further inhibiting the anti-apoptotic protein Bcl-2. ROS can also trigger the nuclear translocation of FOXO3 through oxidative stress, enhancing its transcriptional activity. This, in turn, activates the downstream gene BCL6, which inhibits the anti-apoptotic protein Bcl-XL, promoting apoptosis [30, 32–38]. This ultimately promotes cell death through the mitochondrial apoptotic pathway (Fig. 4E).

Based on these findings, we further examined the changes in apoptosis-related proteins. The results showed that TTK knockdown increased the expression of pro-apoptotic proteins (Bax, Cyt C, and Cleaved-Caspase-3) and decreased the expression of anti-apoptotic proteins (Bcl-XL and Bcl-2). Notably, the mtROS scavenger Tempo significantly reversed these changes induced by TTK knockdown (Fig. 4F). Additionally, Tempo effectively alleviated the increased apoptosis and growth inhibition caused by TTK knockdown (Fig. 4G, H). In summary, these results suggest that by inhibiting mitophagy and increasing mtROS levels, TTK knockdown promotes BC cells apoptosis through the mitochondrial apoptotic pathway.

### TTK promotes mitophagy by regulating the phosphorylation of ULK1 at Ser477

**Fig. 5:**
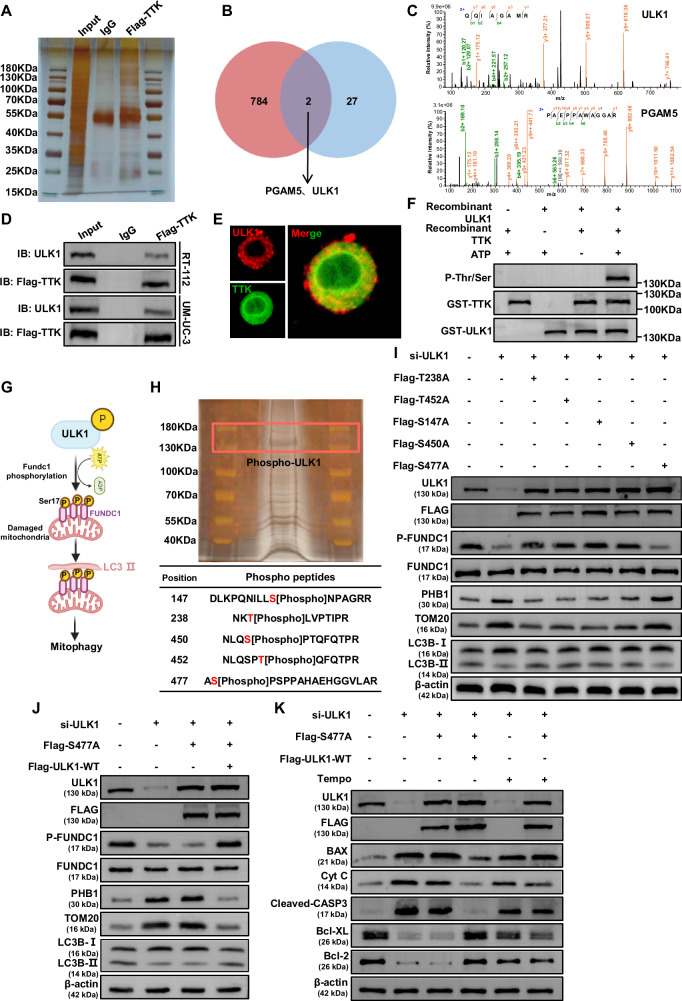
TTK promotes mitophagy by mediating ULK1 Ser477 phosphorylation. **A** Silver staining showing proteins pulled down by Flag-TTK from RT-112 cell lysates. Different bands on the silver-stained gel were excised and assessed by mass spectrometry (MS). **B** Venn diagram displaying the overlap between proteins pulled down by Flag-TTK identified by MS and proteins related to mitophagy. **C** Peptide maps of ULK1 and PGAM5 pulled down by Flag-TTK. **D** Co-immunoprecipitation of Flag-TTK in RT-112 and UM-UC-3 cells using an anti-Flag monoclonal antibody. Co-immunoprecipitated ULK1 was detected with an anti-ULK1 antibody. Co-immunoprecipitated Flag-TTK was detected with an anti-Flag antibody. **E** Immunofluorescence staining assay indicated the co-localization of TTK and ULK1 in RT-112 Cells. (Scale bar: 5 μm). **F** In vitro incubation of recombinant active TTK kinase with purified recombinant ULK1 fusion protein in kinase buffer and ATP for 0.5 hours. Phosphorylated threonine/serine, TTK, and ULK1 proteins were detected by Western blot. **G** Schematic diagram illustrating the activation of mitophagy by phosphorylated ULK1 through the phosphorylation of FUNDC1 at the Ser17 site. **H** Silver staining revealing phosphorylated ULK1 protein in the in vitro kinase assay, with phosphorylation sites on ULK1 being identified by MS. **I** Western blot analysis of mitophagy-related protein levels in RT-112 cells with ULK1 knockdown, transfected with plasmids encoding Flag-tagged ULK1 proteins with different phosphorylation site mutations (T238A, T452A, S147A, S450A, and S477A). **J** Western blot analysis of mitophagy-related protein levels in RT-112 cells with ULK1 knockdown, transfected with plasmids encoding Flag-tagged ULK1 protein with S477A phosphorylation site mutation, followed by transfection with a plasmid encoding Flag-tagged wild-type ULK1 (Flag-ULK1-WT). **K** Western blot analysis of apoptosis-related proteins in RT-112 cells with ULK1 knockdown or ULK1 S477A mutation, followed by further assessment of apoptosis-related protein expression after treatment with the mtROS scavenger Tempo.

To explore the mechanisms by which TTK regulates mitophagy in BC, we performed IP-MS to identify key molecules. Flag-TTK was immunoprecipitated from RT-112 cells using a Flag monoclonal antibody, and co-immunoprecipitated proteins were visualized by silver staining (Fig. 5A) and identified by mass spectrometry (Supplementary Table S4). By intersecting these proteins with key molecules involved in mitophagy (Supplementary Table S5), we identified PGAM5 and ULK1 as potential regulatory proteins of TTK (Fig. 5B, C). Given that TTK is a serine/threonine kinase, primarily catalyzing protein phosphorylation, and that ULK1 requires phosphorylation for activation in mitophagy [39, 40], we speculatively took ULK1 as a key interaction partner for further study.

Co-IP confirmed the interaction between TTK and ULK1. ULK1 was precipitated by Flag-TTK beads in RT-112 but not by control beads (IgG), and the endogenous TTK and ULK1 also interacted in UM-UC-3 (Fig. 5D). Additionally, immunofluorescence confirmed that TTK and ULK1 colocalize in the cytoplasm of BC cells (Fig. 5E).

In vitro kinase assays demonstrated that recombinant human TTK kinase was able to phosphorylate recombinant human ULK1 in the presence of ATP, significantly increasing its serine/threonine phosphorylation levels. Notably, our results showed no evidence of ULK1 autophosphorylation in the presence of ATP (Fig. 5F).

The ULK1/FUNDC1-mediated mitophagy pathway is a crucial cellular mechanism for maintaining mitochondrial health through the removal of damaged mitochondria [41, 42]. In this process, ULK1 undergoes phosphorylation and activation, which in turn leads to the phosphorylation of FUNDC1 at Ser17. This phosphorylation event triggers mitophagy, marking damaged mitochondria for recognition and subsequent transport to lysosomes for degradation. This pathway is critical for maintaining mitochondrial homeostasis, regulating apoptosis, and responding to cellular stress [43–47] (Fig. 5G).

To further investigate whether TTK-mediated phosphorylation of ULK1 activates mitophagy and to identify which phosphorylation sites on ULK1 play a critical role, we first visualized phosphorylated ULK1 from in vitro kinase assays using silver staining and then performed mass spectrometry analysis. The analysis identified five phosphorylation sites on ULK1: Thr238, Thr452, Ser147, Ser450, and Ser477 (Fig. 5H). These sites are highly conserved across species, suggesting their potential importance in regulating ULK1 function (Supplementary Table S6). To determine which phosphorylation sites are crucial for ULK1 activation and the induction of mitophagy, we mutated these serine or threonine residues to alanine, generating non-phosphorylatable mutants, including Flag-ULK1-T238A, Flag-ULK1-T452A, Flag-ULK1-S147A, Flag-ULK1-S450A, and Flag-ULK1-S477A. The results showed that mutation at the Ser477 site of ULK1 significantly reduced FUNDC1 Ser17 phosphorylation, decreased LC3B-II expression, and increased levels of the outer mitochondrial membrane protein TOM20 and inner mitochondrial membrane protein PHB1 (Fig. 5I and Supplementary Fig. S5A), indicating impaired mitophagy. Further experiments demonstrated that wild-type Flag-ULK1 rescued the mitophagy inhibition caused by the ULK1 Ser477 mutation (Fig. 5J and Supplementary Fig. S5B). This finding was further validated by mt-Keima reporter analysis. In cells with ULK1 knockdown or the ULK1 Ser477 mutation, fluorescence excited at 550 nm was significantly reduced, while fluorescence excited at 440 nm was increased, indicating impaired mitophagy. Conversely, the introduction of wild-type Flag-ULK1 significantly reversed these effects, restoring normal levels of mitophagy (Supplementary Fig. S5C). Further analysis revealed that ULK1 knockdown or the ULK1 Ser477 mutation led to increased expression of pro-apoptotic proteins (Bax, Cyt C, and Cleaved-Caspase-3), decreased expression of anti-apoptotic proteins (Bcl-XL and Bcl-2), which ultimately increased apoptosis and inhibited cell proliferation. The mtROS scavenger Tempo significantly mitigated these changes (Fig. 5K, Supplementary Fig. S5D, E). Together, these findings suggest that TTK directly binds to and phosphorylates ULK1 at Ser477, activating the ULK1/FUNDC1-mediated mitophagy pathway, and inhibiting mitochondrial apoptosis in BC cells. Targeting TTK may, therefore, provide a promising therapeutic strategy to induce apoptosis and suppress BC progression.

### TTK inhibition promotes ULK1 exon 5 skipping and nonsense-mediated mRNA decay (NMD)

**Fig. 6:**
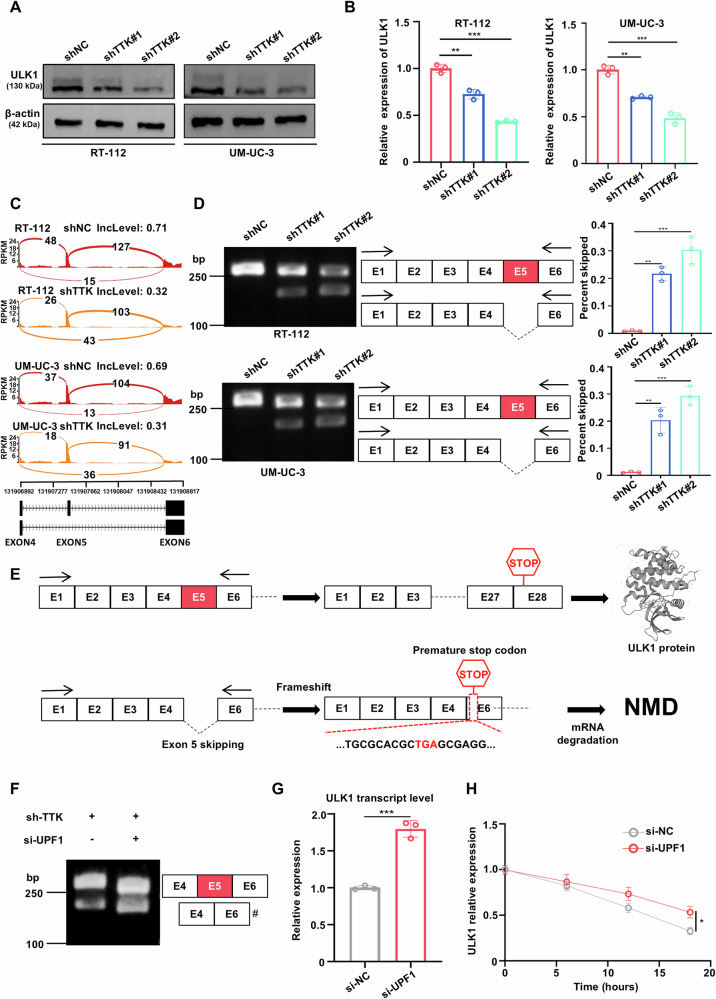
TTK knockdown promotes ULK1 Exon 5 skipping leading to alternative splicing-nonsense-mediated mRNA decay (AS-NMD). **A** Western blot analysis of ULK1 protein expression in RT-112 and UM-UC-3 cells stably transfected with shNC, shTTK#1, or shTTK#2. **B** qRT-PCR determination of ULK1 mRNA expression in RT-112 and UM-UC-3 cells stably transfected with shNC, shTTK#1, or shTTK#2. **C** Sashimi plot showing ULK1 exon 5 skipping in RT-112 and UM-UC-3 cells stably transfected with shNC (top) or shTTK (bottom). **D** Representative gel images from RT-PCR showing normal and abnormal splicing products of ULK1 mRNA in RT-112 and UM-UC-3 cells transfected with shNC, shTTK#1, or shTTK#2. Quantification of the percentage of RT-PCR products with exon 5 skipping among total ULK1 transcripts. **E** Schematic diagram illustrating how ULK1 exon 5 skipping may trigger NMD. Inclusion of exon 5 results in a functional protein with a termination codon in exon 28. Skipping exon 5 causes a frameshift, generating a premature termination codon at the start of exon 6, 12 nucleotides upstream of the last exon-exon junction. **F**, **G** RT-PCR detection of different isoforms of the indicated transcripts in RT-112 cells when NMD was inhibited. Cells were transfected with si-UPF1 or not after TTK knockdown. A number sign (#) indicates the NMD-sensitive isoform (**F**). Total levels of ULK1 transcripts in RT-112 cells after specific treatments were analyzed by qRT-PCR (**G**). **H** qRT-PCR measurement of ULK1 mRNA expression in RT-112 cells transfected with si-NC or si-UPF1 and treated with 10 μg/ml actinomycin D. Data are presented as mean ± SD from three independent experiments. *P < 0.05; **P < 0.01; ***P < 0.001.

In studying the effect of TTK on ULK1 phosphorylation, we unexpectedly discovered that TTK knockdown reduced ULK1 protein expression (Fig. 6A and Supplementary Fig. S6A). Further, qRT-PCR analysis revealed that TTK knockdown also decreased ULK1 mRNA levels (Fig. 6B and Supplementary Fig. S6B), consistent with the mRNA expression trends of TTK and ULK1 observed in the TCGA BLCA dataset (Supplementary Fig. S6C). To investigate the underlying cause of this effect, we performed an RNA-Seq on RT-112, UM-UC-3, and 5637 cells with TTK knockdown. RNA-Seq results showed that TTK knockdown promoted exon 5 skipping in ULK1 mRNA (Fig. 6C and Supplementary Fig. S6D).

To further confirm whether the splicing of ULK1 pre-mRNA was regulated by TTK knockdown, we performed RT-PCR on the 28 exons of human ULK1. Agarose gel electrophoresis of PCR products, using primers covering exons 1 and 6 of human ULK1 mRNA, showed a newly generated shorter transcript of approximately 200 bp in TTK knockdown BC cells (Fig. 6D and Supplementary Fig. S6E), with no significant differences observed in other regions (Supplementary Fig. S6F). RT-PCR using primers covering exons 1 to 4 of human ULK1 mRNA found no significant differences between TTK knockdown and control groups (Supplementary Fig. S6G). Therefore, we confirmed that TTK knockdown did induce ULK1 exon 5 skipping.

Additionally, the Kyoto Encyclopedia of Genes and Genomes (KEGG) and Gene Ontology (GO) enrichment analyses indicated that TTK was closely associated with apoptosis and mRNA processing (Supplementary Fig. S6H, I), supporting our previous findings.

Sequence analysis exhibited that skipping of ULK1 exon 5 resulted in a frameshift, creating a premature termination codon at the start of exon 6, which is 12 nucleotides upstream of the last exon-exon junction. Transcripts with this termination codon were predicted to be degraded via the NMD pathway (Fig. 6E). To verify whether ULK1 exon 5 skipping transcripts are degraded by NMD, we silenced the core NMD factor UPF1 and found that the silencing inhibited NMD and significantly increased the levels of exon 5 skipping transcripts, indicating that exon 5 skipping produces an NMD-sensitive isoform (Fig. 6F). Consistently, ULK1 exon 5 skipping transcripts were stabilized, and total ULK1 transcript levels increased in response to UPF1 knockdown (Fig. 6G). Furthermore, in TTK knockdown RT-112 cells, UPF1 knockdown increased the RNA half-life of exon 5 skipping transcripts after actinomycin D treatment, confirming the NMD sensitivity of these transcripts (Fig. 6H). Overall, TTK knockdown promotes ULK1 exon 5 skipping, triggering NMD-mediated transcript degradation.

### TTK-mediated SRSF3 Ser108 phosphorylation prevents ULK1 exon 5 skipping

**Fig. 7:**
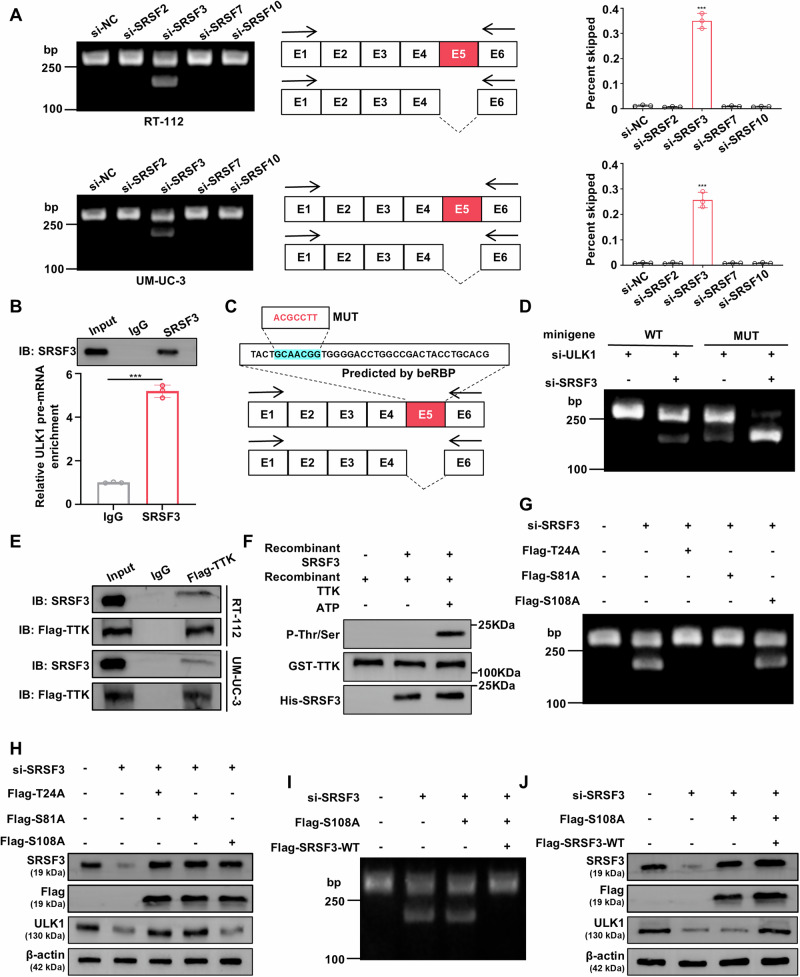
TTK inhibits ULK1 exon 5 skipping by mediating SRSF3 Ser108 phosphorylation. **A** Representative gel images from RT-PCR experiments showing normal and abnormal splicing products of ULK1 mRNA in RT-112 and UM-UC-3 cells transfected with negative control siRNA (si-NC), si-SRSF2, si-SRSF3, si-SRSF7 or si-SRSF10. Quantification of the percentage of RT-PCR products with exon 5 skipping among total transcripts. **B** RNA immunoprecipitation (RIP) confirmed the interaction between SRSF3 and ULK1 pre-mRNA. **C** Computational prediction of SRSF3 binding sites (blue regions) on ULK1 pre-mRNA using beRBP. The blue-highlighted section indicates the predicted binding sequences of SRSF3 with ULK1 exon 5, and the red font indicates the random mutation. **D** RT-112 cells were first transfected with si-ULK1 to knock down endogenous ULK1, followed by transfection with 1 µg GFP-ULK1 minigene wild type (WT minigene) or GFP-ULK1 minigene mutant (Mut minigene), in which the GCAACGG sequence was mutated to ACGCCTT, and then co-transfected with or without si-SRSF3. Representative gel images of GFP-ULK1 transcripts by RT-PCR. **E** Co-immunoprecipitation of Flag-TTK in RT-112 and UM-UC-3 cells using an anti-Flag monoclonal antibody. Co-immunoprecipitated SRSF3 was detected with an anti-SRSF3 antibody. Co-immunoprecipitated Flag-TTK was detected with an anti-Flag antibody. **F** In vitro incubation of active recombinant TTK kinase with purified recombinant SRSF3 fusion protein in kinase buffer and ATP for 0.5 hours. Phosphorylated threonine/serine, TTK, and SRSF3 proteins were detected by Western blot. RT-PCR showing representative gel images of normal and abnormal splicing products of ULK1 mRNA (**G**) and Western blot analysis of ULK1 protein expression (**H**) in RT-112 cells with SRSF3 knockdown, transfected with plasmids encoding Flag-tagged SRSF3 proteins with different phosphorylation site mutations (T24A, S18A, and S108A). Representative gel images of normal and abnormal splicing products of ULK1 mRNA by RT-PCR (**I**) and Western blot results of ULK1 protein expression (**J**) in RT-112 cells with SRSF3 knockdown, transfected with plasmids encoding Flag-tagged SRSF3 protein with S108A phosphorylation site mutation, followed by transfection with plasmid encoding wild-type Flag-tagged SRSF3 (Flag-SRSF3-WT). Data are presented as mean ± SD from three independent experiments. ***P < 0.001.

Since TTK is a serine/threonine kinase primarily involved in catalyzing protein phosphorylation rather than splicing, we hypothesized that TTK may regulate pre-mRNA splicing by modulating a splicing-related protein. Our IP-MS results identified the SRSF family as potential candidates. Mass spectrometry suggested that TTK might interact with members of the SR protein family, including SRSF2, SRSF3, SRSF7, and SRSF10 (Supplementary Fig. S7A). We proceeded to knock down each of these proteins in BC cells and evaluated their role in ULK1 pre-mRNA splicing using RT-PCR. Apart from the discovery of a newly generated shorter transcript in the primer set covering exons 1 and 6 of human ULK1 mRNA, no significant differences were observed in other regions (Fig. 7A and Supplementary Fig. S7B), including the primer set covering exons 1 and 4 of human ULK1 mRNA (Supplementary Fig. S7C). These results suggest that SRSF3 knockdown enhances ULK1 exon 5 skipping.

To further confirm the role of SRSF3 in the regulation of ULK1 alternative splicing, we performed RIP and found the interaction between SRSF3 and ULK1 pre-mRNA (Fig. 7B). Splicing factors regulate the inclusion or exclusion of alternative exons by recognizing different RNA sequences [48]. We predicted the potential SRSF3 binding site sequence in exon 5 of ULK1 pre-mRNA using beRBP, with the predicted sequence being GCAACGG (Fig. 7C). To verify whether the interaction between SRSF3 and exon 5 of ULK1 pre-mRNA is responsible for the inclusion of exon 5 in ULK1 mRNA, we randomly altered the binding sequence using an online random mutagenesis tool (https://www.detaibio.com/sms2/mutate_dna.html). We then constructed a GFP-ULK1 minigene mutant in which the GCAACGG sequence was mutated to ACGCCTT (Mut minigene). Expression of the mutant minigene in RT-112 cells resulted in significantly reduced exon 5 inclusion compared to the wild-type minigene, mimicking the effect of SRSF3 knockdown (Fig. 7D). These results indicate that this sequence acts as an exon splicing enhancer (ESE) region, and SRSF3 binding to this ESE is essential for the inclusion of exon 5 in the full-length ULK1 mRNA.

Next, we examined the interaction between TTK and SRSF3. Co-IP results confirmed the interaction between TTK and SRSF3 in BC cells (Fig. 7E). In vitro kinase assays showed that recombinant human SRSF3 could be phosphorylated by recombinant human TTK kinase, thus increasing serine/threonine phosphorylation levels (Fig. 7F). Mass spectrometry detection of recombinant SRSF3 identified Thr24, Ser81, and Ser108 as the phosphorylation sites modified by TTK (Supplementary Fig. S8A). These sites are highly conserved across different species (Supplementary Table S7). To investigate the functionality of these phosphorylation sites, we mutated these serine or threonine residues to alanine. The results showed that SRSF3 knockdown or SRSF3 Ser108 mutation significantly increased ULK1 exon 5 skipping (Fig. 7G). qRT-PCR and western blot analysis demonstrated that SRSF3 knockdown or Ser108 mutation also reduced ULK1 mRNA and protein levels (Fig. 7H and Supplementary Fig. S8B), consistent with the correlation between SRSF3 and ULK1 mRNA expression observed in the TCGA BLCA dataset (Supplementary Fig. S8C). Expression of wild-type Flag-SRSF3 rescued the decrease in ULK1 mRNA and protein levels caused by the SRSF3 Ser108 mutation (Fig. 7I, J, Supplementary Fig. S8D, E). These results suggest that TTK phosphorylates SRSF3 at Ser108, preventing ULK1 exon 5 skipping, thus regulating ULK1 expression and ensuring proper mRNA processing.

## Discussion

Kinases play key roles in cellular signaling and are important targets in cancer research. Our study revealed the critical role of TTK in the development and progression of BC, suggesting its potential as a therapeutic target. We found that TTK is significantly overexpressed in BC cells and tissues, correlating with poor prognosis, which aligns with its established role as an oncogene in other cancers [14, 49–53]. Importantly, beyond its established role in promoting cell proliferation, we uncovered a novel function of TTK in regulating mitochondrial quality control and apoptosis via its involvement in mitophagy.

A key finding in our study is that TTK enhances mitophagy, a critical cellular process for clearing damaged mitochondria. Impaired mitophagy leads to the accumulation of dysfunctional mitochondria, which in turn triggers cellular stress and apoptosis [54]. Using transmission electron microscopy, mt-Keima reporter assays, and COX8-EGFP-mCherry plasmid imaging, we found that TTK knockdown significantly impaired mitophagy. This was further evidenced by elevated mtDNA levels and increased expression of mitochondrial membrane proteins (TOM20 and PHB1). Additionally, reduced LC3B puncta and decreased co-localization of MitoTracker Green and LysoTracker Red indicated a defect in mitochondrial degradation. These results strongly suggest that TTK promotes the clearance of dysfunctional mitochondria.

Impaired mitophagy often leads to excessive mtROS production, which causes oxidative damage, disrupts mitochondrial membrane potential, and activates apoptotic pathways [28, 30–32]. TTK knockdown significantly increased mtROS levels, as shown by MitoSOX, MitoP/MitoB, DHE staining, and DCFH-DA flow cytometry. This was accompanied by disrupted mitochondrial membrane potential (evidenced by JC-1 and TMRE assays) and increased apoptosis, marked by elevated pro-apoptotic proteins (Bax, Cyt C, Cleaved-Caspase-3) and decreased anti-apoptotic proteins (Bcl-2, Bcl-XL). Treatment with the mtROS scavenger Tempo reversed these effects, confirming that mtROS accumulation plays a critical role in TTK knockdown-induced apoptosis. The effects of TTK knockdown on mtROS and apoptosis were time-dependent, with significant increases observed at later time points. These findings highlight the progressive activation of apoptotic pathways due to mitochondrial dysfunction and underscore TTK’s crucial role in maintaining mitochondrial homeostasis.

At the molecular level, we identified ULK1 as a key target of TTK in regulating mitophagy. Our findings show that TTK directly phosphorylates ULK1 at Ser477, a critical site for ULK1 activation. Phosphorylation of ULK1 at Ser477 enhances its ability to activate the ULK1/FUNDC1-mediated mitophagy pathway. ULK1 knockdown or mutation of ULK1 Ser477 resulted in impaired mitophagy, characterized by reduced FUNDC1 Ser17 phosphorylation and mitochondrial protein accumulation, leading to defective mitochondrial turnover. These findings underscore the essential role of ULK1 Ser477 phosphorylation in the activation of mitophagy by TTK.

In addition to post-translational regulation, we also discovered that TTK modulates ULK1 expression through alternative splicing. TTK knockdown promoted exon 5 skipping in ULK1 pre-mRNA, leading to the production of truncated, NMD-sensitive transcripts. This reduction in full-length ULK1 mRNA further impaired mitophagy and promoted apoptosis. Through IP-MS and RT-PCR, we confirmed that TTK regulates ULK1 splicing, with SRSF3 acting as a critical splicing factor. TTK phosphorylates SRSF3 at Ser108, preventing ULK1 exon 5 skipping and ensuring proper ULK1 expression.

**Fig. 8:**
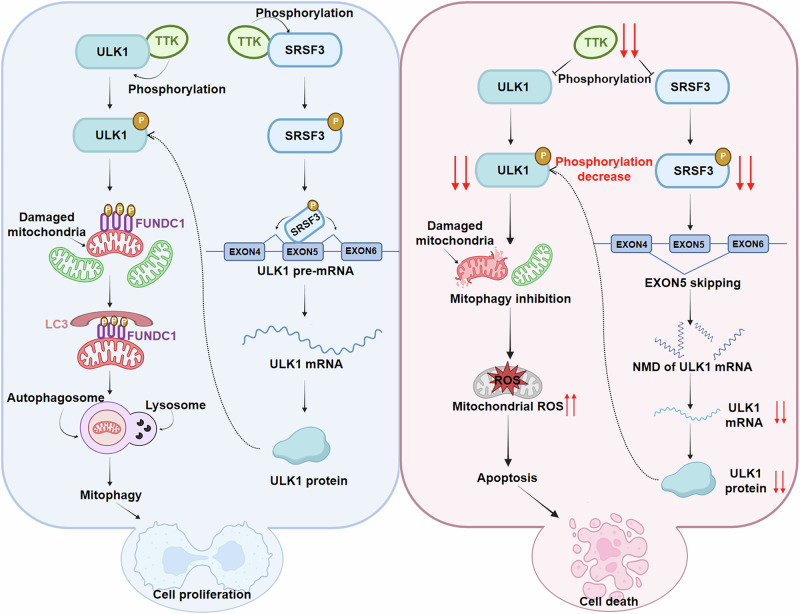
Schematic diagram of TTK regulation of mitophagy. In BC cells with high TTK expression, TTK phosphorylates ULK1 at the Ser477 site, which subsequently phosphorylates FUNDC1 at the Ser17 site, inducing mitophagy. TTK also phosphorylates SRSF3 at the Ser108 site, preventing ULK1 exon 5 skipping and maintaining ULK1 mRNA stability, thereby increasing ULK1 protein expression. These processes promote high levels of mitophagy, enhancing BC cells survival under conditions such as oxidative stress or hypoxia. When TTK is knocked down, phosphorylation of ULK1 at Ser477 is reduced, inhibiting mitophagy and leading to the accumulation of damaged mitochondria and excessive mtROS production, promoting mitochondrial apoptosis. TTK knockdown also facilitates ULK1 exon 5 skipping, triggering the NMD pathway, reducing ULK1 mRNA and protein levels, further decreasing mitophagy, and accelerating BC cells mitochondrial apoptosis.

Taken together, our findings reveal that TTK regulates mitophagy in BC cells through a dual mechanism: by phosphorylating ULK1 at Ser477 to activate mitophagy and by modulating ULK1 pre-mRNA splicing through SRSF3 Ser108 phosphorylation. Disruption of these processes leads to mitochondrial dysfunction, mtROS accumulation, and apoptosis, highlighting the critical role of TTK in maintaining mitochondrial homeostasis in BC cells (Fig. 8).

In conclusion, our study identifies TTK as a crucial regulator of mitophagy and apoptosis in BC, through its dual regulation of ULK1 at both the splicing and phosphorylation levels. Disrupting these processes leads to mitochondrial dysfunction, excessive mtROS accumulation, and apoptosis, emphasizing TTK’s significant role in BC progression. From a therapeutic perspective, targeting TTK could promote BC cell apoptosis by impairing mitophagy and activating the mitochondrial apoptosis pathway. Furthermore, the TTK inhibitor CFI-402257 effectively suppressed tumor growth in vivo, suggesting that targeting TTK may offer a promising approach for BC treatment. Mitophagy dysregulation is also linked to chemotherapy and targeted therapy resistance [55–57], and inhibiting TTK may sensitize drug-resistant BC cells to these therapies, potentially improving the efficacy of conventional treatments. This combined approach not only offers a novel therapeutic strategy for BC but also has broader implications for cancers exhibiting abnormal mitophagy and therapy resistance, providing a solid foundation for future clinical applications.

## Supplementary information

Supplementary Figures

Supplementary Tables

Uncropped blot and gel images

## Data Availability

The datasets generated or analyzed during the current study are available in the TCGA BLCA repository (https://portal.gdc.cancer.gov/) and the GEO database (https://www.ncbi.nlm.nih.gov/geo/). The RNA-Seq data generated or analyzed during the current study are available from the corresponding author on reasonable request.
