# Correction: TTK promotes mitophagy by regulating ULK1 phosphorylation and pre-mRNA splicing to inhibit mitochondrial apoptosis in bladder cancer

**DOI:** 10.1038/s41418-025-01571-y

**Published:** 2025-09-01

**Authors:** Kang Chen, Jinyu Chen, Yukun Cong, Qingliu He, Chunyu Liu, Jiawei Chen, Haoran Li, Yunjie Ju, Liang Chen, Yarong Song, Yifei Xing

**Affiliations:** 1 Department of Urology, Union Hospital, Tongji Medical College, Huazhong University of Science and Technology, Wuhan, China; 2 Department of Urology, The Second Affiliated Hospital of Fujian Medical University, Quanzhou, China

**Keywords:** Autophagy, Oncogenes, Kinases

Correction to: *Cell Death & Differentiation* 10.1038/s41418-025-01492-w, published online 23 April 2025

An error has been identified in the originally published version of this article. Specifically, the image for the shTTK#2 panel in Figure 3E was inadvertently duplicated from the shTTK#1 panel in Figure 3H. This error occurred due to a file copying mistake during the manual archiving of the original grayscale image files. The duplicated original grayscale file was subsequently subjected to different pseudo-coloring. This correction does not affect the interpretation of the figure or the conclusions of the study.

**Original Figure 3** 
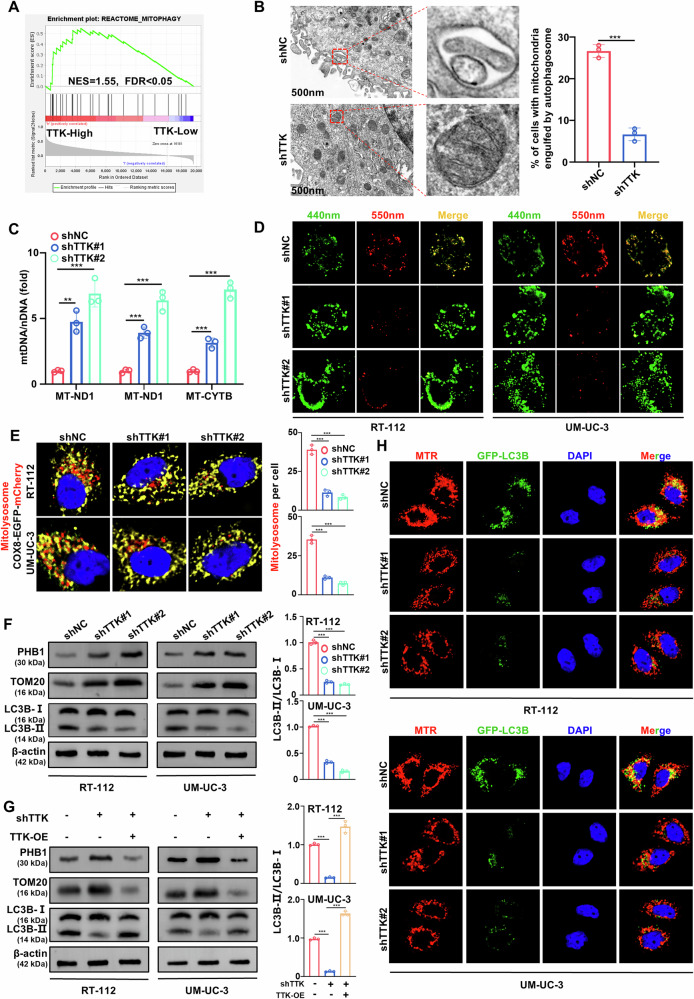


**Amended Figure 3** 
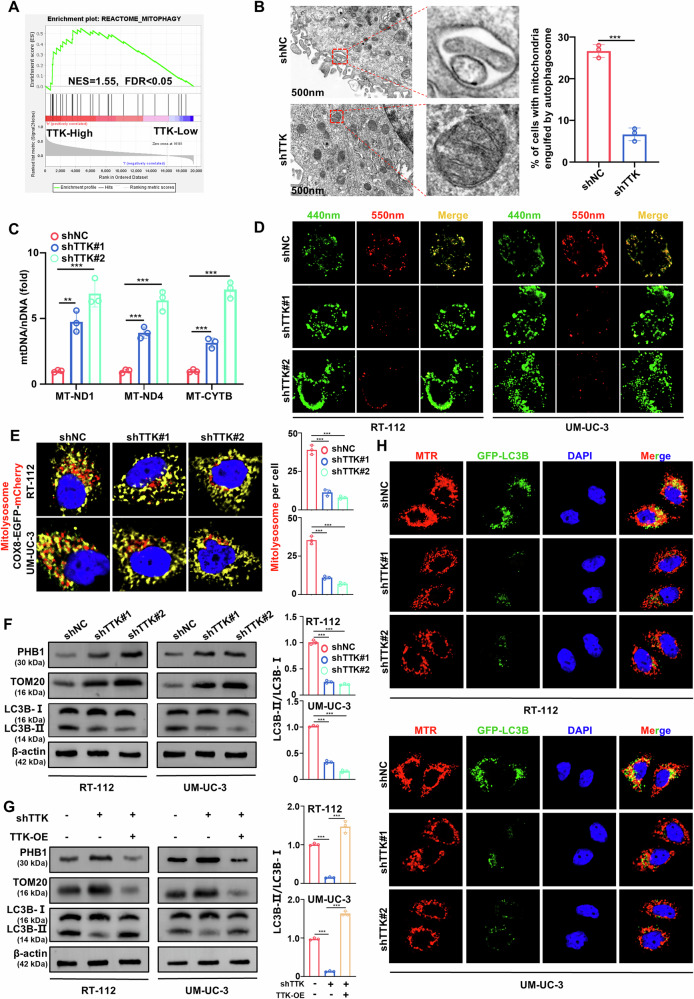


The original article has been corrected.

