# Supplementary material for: TTK promotes mitophagy by regulating ULK1 phosphorylation and pre-mRNA splicing to inhibit mitochondrial apoptosis in bladder cancer
Source: Cell Death Differ. 2025 Apr 23;32(9):1691–706. doi: 10.1038/s41418-025-01492-w (PMC12432130; doi:10.1038/s41418-025-01492-w)
Supplement: Supplementary file 1 — Supplementary Figures [file 41418_2025_1492_MOESM1_ESM.docx]

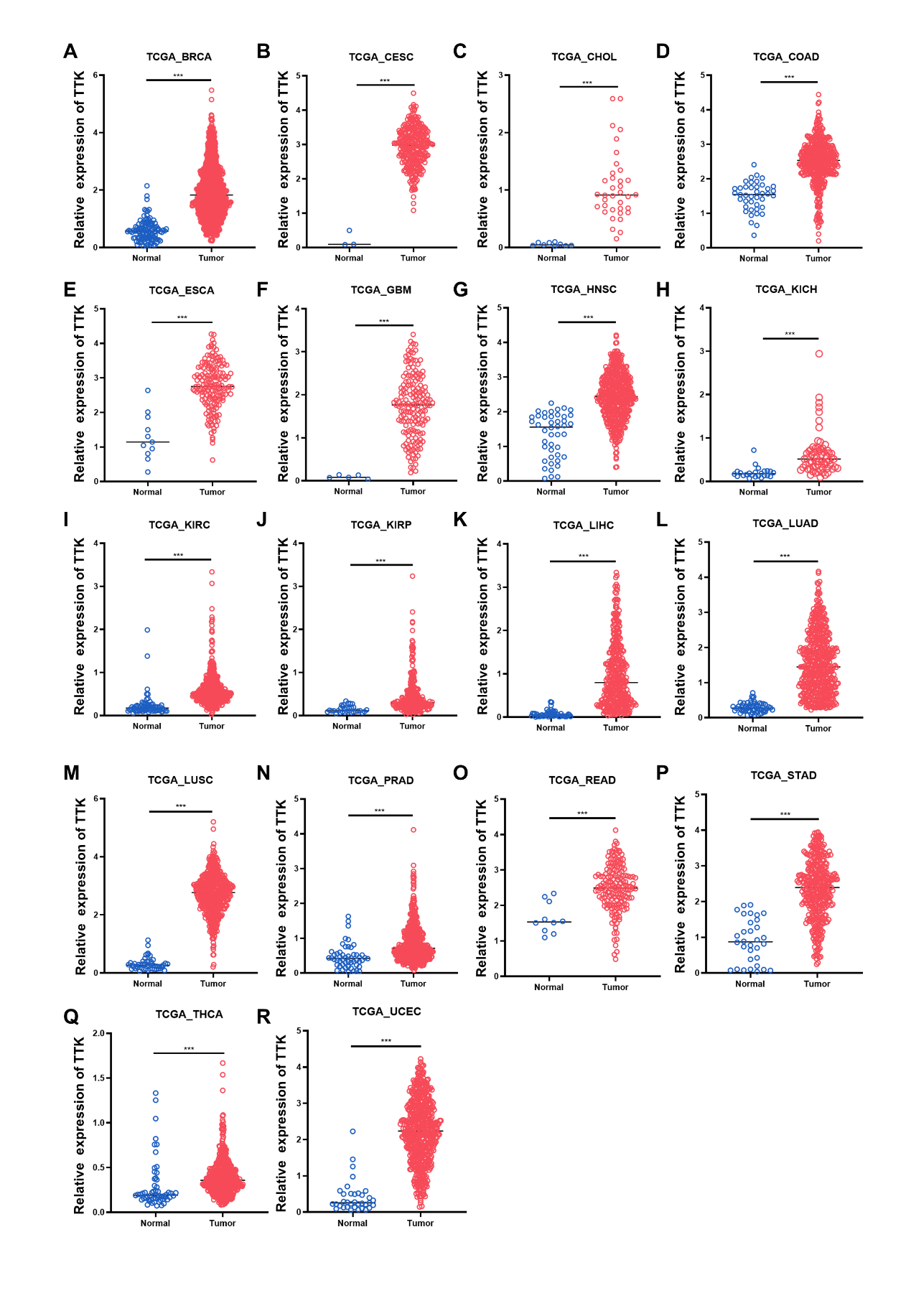


**Supplementary Figure S1. The TCGA Database Shows TTK is Upregulated in Tumor Tissues Compared to Adjacent Non-Tumor Tissues in Several Solid Cancers.**

(A) Breast invasive carcinoma (BRCA). (B) Cervical squamous cell carcinoma and endocervical adenocarcinoma (CESC). (C) Cholangiocarcinoma (CHOL). (D) Colon adenocarcinoma (COAD). (E) Esophageal carcinoma (ESCA). (F) Glioblastoma multiforme (GBM). (G) Head and Neck squamous cell carcinoma (HNSC). (H) Kidney Chromophobe (KICH). (I) Kidney renal clear cell carcinoma (KIRC). (J) Kidney renal papillary cell carcinoma (KIRP). (K) Liver hepatocellular carcinoma (LIHC). (L) Lung adenocarcinoma (LUAD). (M) Lung squamous cell carcinoma (LUSC). (N) Prostate adenocarcinoma (PRAD). (O) Rectum adenocarcinoma (READ). (P) Stomach adenocarcinoma (STAD). (Q) Thyroid carcinoma (THCA). (R) Uterine Corpus Endometrial Carcinoma (UCEC). ****P* < 0.001.


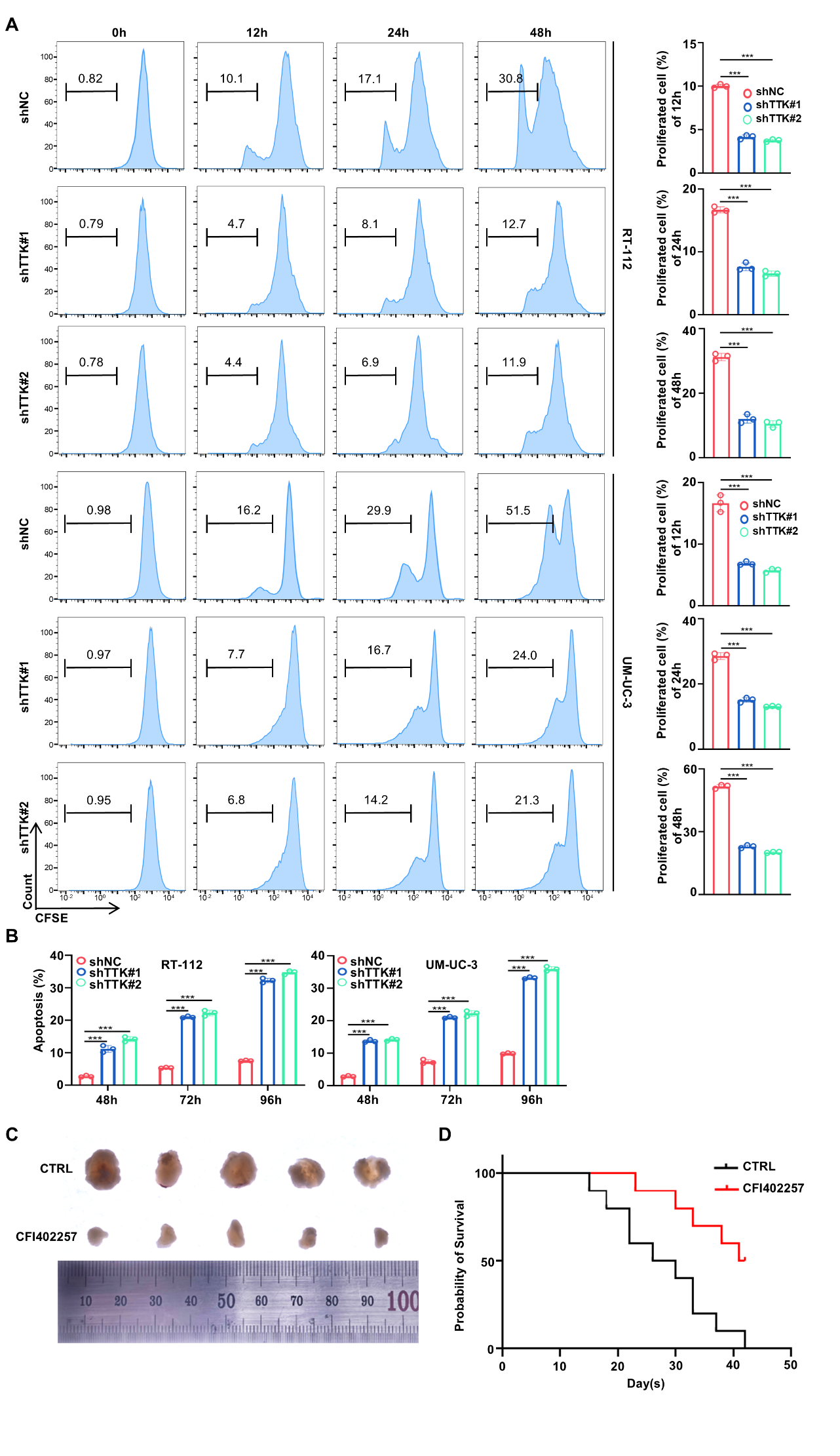


**Supplementary Figure S2. TTK knockdown inhibits proliferation and promotes apoptosis in BC cells.**

(A) CFSE staining was used to assess the proliferation of RT-112 and UM-UC-3 cells stably transfected with shNC, shTTK#1, or shTTK#2 at 0h, 12h, 24h, and 48h. (B) Flow cytometry analysis of apoptosis in RT-112 and UM-UC-3 cells stably transfected with shNC, shTTK#1, or shTTK#2 at 48h, 72h, and 96h. (C) Representative images of xenograft tumors generated by subcutaneous injection of RT-112 cells into nude mice, with or without oral treatment with the TTK inhibitor CFI-402257 (n = 10 mice per group). (D) Survival percentage of tumor-bearing mice treated with vehicle control (CTRL) or CFI-402257 at 6 mg/kg/day. Data are expressed as mean ± SD from three independent experiments. ****P* < 0.001.


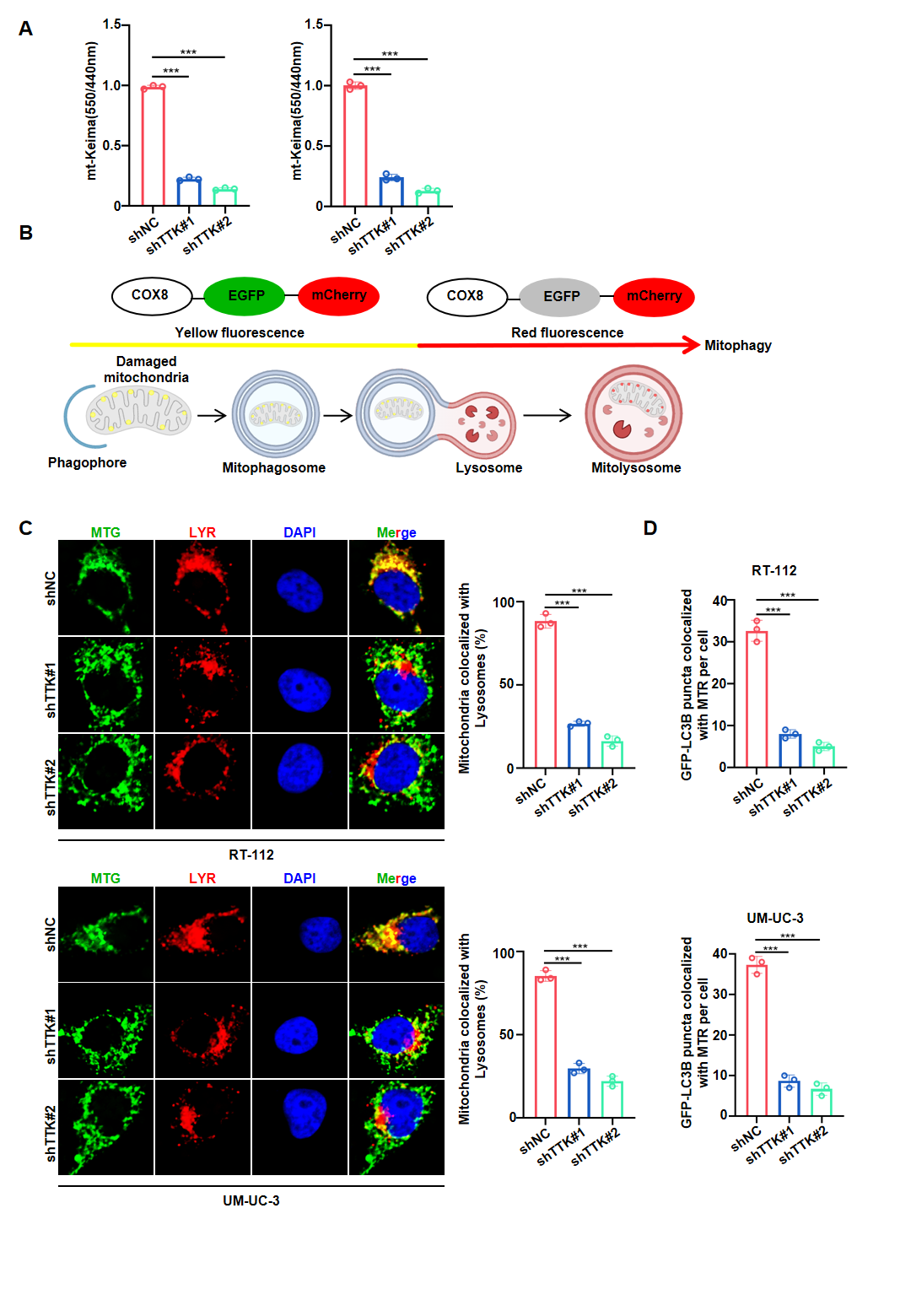


**Supplementary Figure S3. TTK knockdown Suppresses Mitophagy of BC cells.**

(A) Quantification of the mitophagy index (mt-Keima 550/440 nm ratio) in RT-112 and UM-UC-3 cells stably transfected with shNC, shTTK#1, or shTTK#2. (B) Schematic diagram illustrating the use of COX8-EGFP-mCherry plasmid to monitor mitophagy. Under normal conditions, mitochondria exhibit yellow fluorescence due to the overlap of EGFP (green) and mCherry (red). However, when mitochondria are damaged, autophagosomes recognize and encapsulate them, leading to their transport to lysosomes. The fusion of autophagosomes with lysosomes results in the quenching of green fluorescence, and mitochondria trapped in autolysosomes emit only red fluorescence. (C)Immunofluorescence-based quantification of mitophagosomes in RT-112 and UM-UC-3 cells stably transfected with shNC, shTTK#1, or shTTK#2, using MitoTracker Green (MTG) and LysoTracker Red (LYR) to assess mitochondrial-lysosome co-localization. (Scale bar: 5 μm) (D) Quantification of GFP-LC3B and MitoTracker Red (MTR) co-localization puncta in RT-112 and UM-UC-3 cells stably transfected with shNC, shTTK#1, or shTTK#2. Data are expressed as mean ± SD from three independent experiments. ****P* < 0.001.


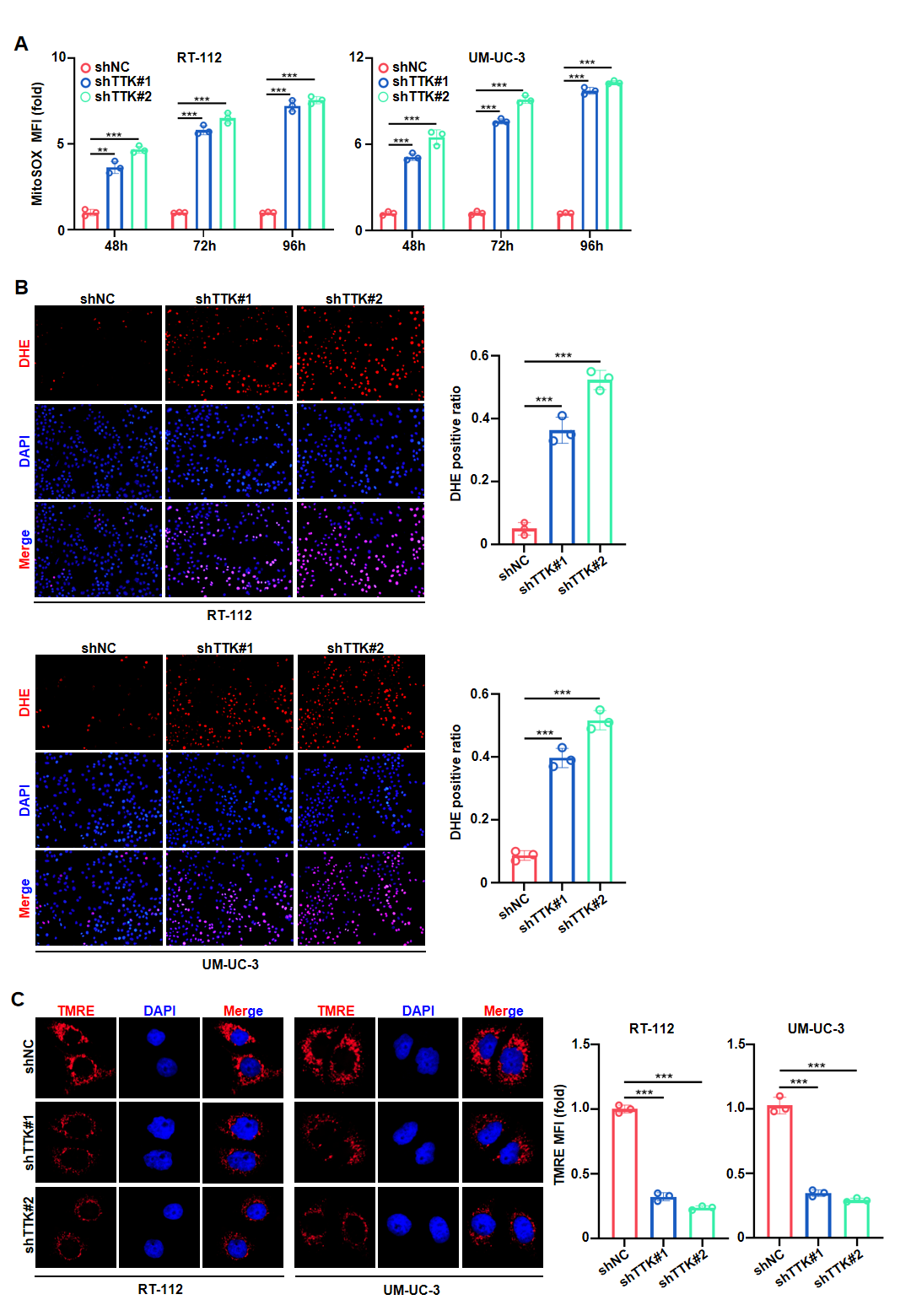


**Supplementary Figure S4. TTK knockdown promotes mtROS accumulation and disrupts mitochondrial membrane potential.**

(A) Quantification of MitoSOX mean fluorescence intensity (MFI) in RT-112 and UM-UC-3 cells stably transfected with shNC, shTTK#1, or shTTK#2 at 48h, 72h, and 96h. (B) Representative DHE staining images and quantification of the DHE-positive ratio in RT-112 and UM-UC-3 cells stably transfected with shNC, shTTK#1, or shTTK#2. (Scale bar: 50 μm). (C) Representative immunofluorescence images of TMRE staining and quantification of TMRE MFI in RT-112 and UM-UC-3 cells stably transfected with shNC, shTTK#1, or shTTK#2. (Scale bar: 10 μm). Data are expressed as mean ± SD from three independent experiments. ***P* < 0.01; ****P* < 0.001.


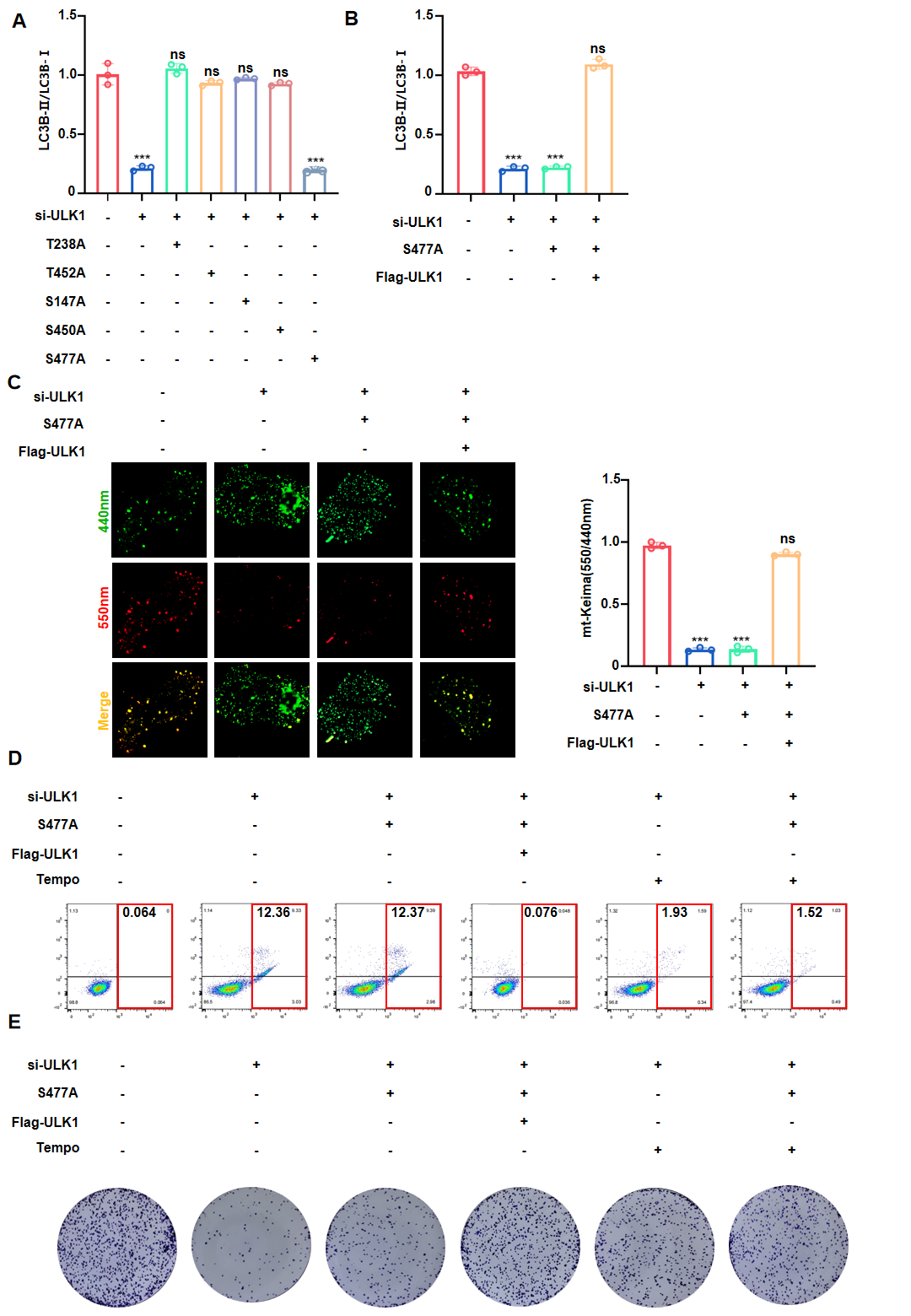


**Supplementary Figure S5. Knockdown of ULK1 or Mutation at the S477 Site Suppresses Mitophagy and Promotes Apoptosis.**

(A-B) Quantification of the fold change in LC3B-II protein levels (LC3B-II/LC3B-I) following ULK1 knockdown or ULK1 S477A mutation (refer to Figure 5I for experimental results in panel A and Figure 5J for panel B). (C) Mt-Keima reporter assay analyzing the effect of ULK1 knockdown or ULK1 S477A mutation on mitophagy in RT-112 cells. Quantification of the mitophagy index (mt-Keima 550/440 nm ratio). (Scale bar: 10 μm). (D) Flow cytometry analysis of the effect of ULK1 knockdown or ULK1 S477A mutation on apoptosis in RT-112 cells, followed by further assessment of apoptosis after treatment with the mtROS scavenger Tempo. (E) Colony formation assay investigating the effect of ULK1 knockdown or ULK1 S477A mutation on the proliferation of RT-112 cells, followed by further assessment of cell proliferation after treatment with the mtROS scavenger Tempo. Data are expressed as mean ± SD from three independent experiments. ns, no significance; ****P* < 0.001.


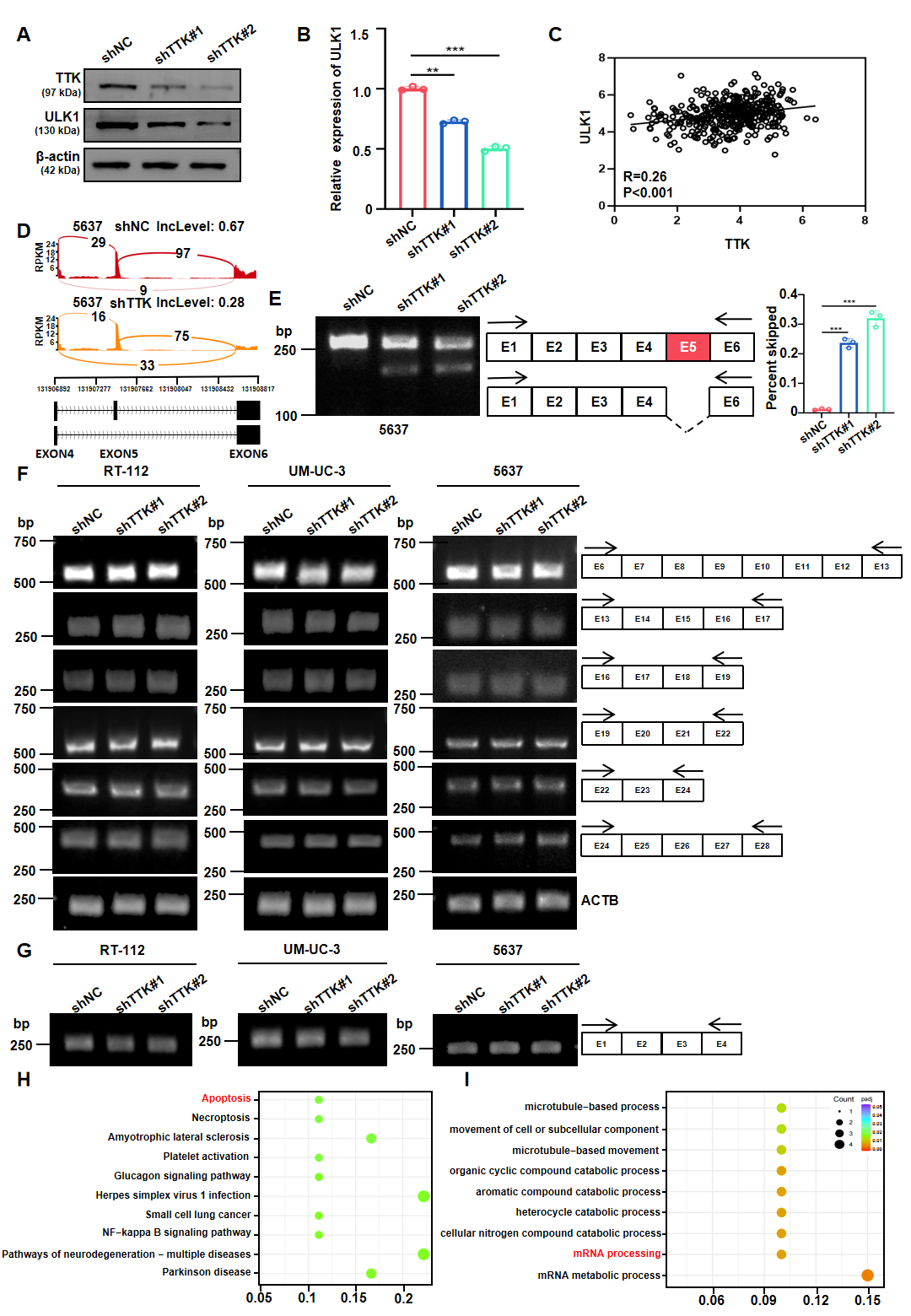


**Supplementary Figure S6. Knockdown of TTK Does Not Affect Other RT-PCR Products of ULK1 mRNA.**

(A) Western blot analysis of ULK1 protein expression in 5637 cells stably transfected with shNC, shTTK#1, or shTTK#2. (B) qRT-PCR analysis of ULK1 mRNA expression in 5637 cells stably transfected with shNC, shTTK#1, or shTTK#2. (C) Pearson correlation analysis showing a significant positive correlation between ULK1 and TTK expression in the TCGA BLCA dataset.

(D) Sashimi plot showing ULK1 exon 5 skipping in 5637 cells stably transfected with shNC (top) or shTTK (bottom). (E) Representative gel images from RT-PCR showing normal and abnormal splicing products of ULK1 mRNA in 5637 cells transfected with shNC, shTTK#1, or shTTK#2. Quantification of the percentage of RT-PCR products with exon 5 skipping among total ULK1 transcripts. (F and G) Representative gel images from RT-PCR experiments showing ULK1 mRNA splicing products in RT-112, UM-UC-3, and 5637 cells transfected with shNC, shTTK#1, or shTTK#2. Each product was confirmed by DNA sequencing. β-actin (ACTB) was used as an internal control. (H and I) KEGG pathway (H) and GO biological process (I) enrichment analysis of differentially downregulated genes in RNA-seq data from TTK knockdown RT-112. Data are expressed as mean ± SD from three independent experiments. ***P* < 0.01; ****P* < 0.001.


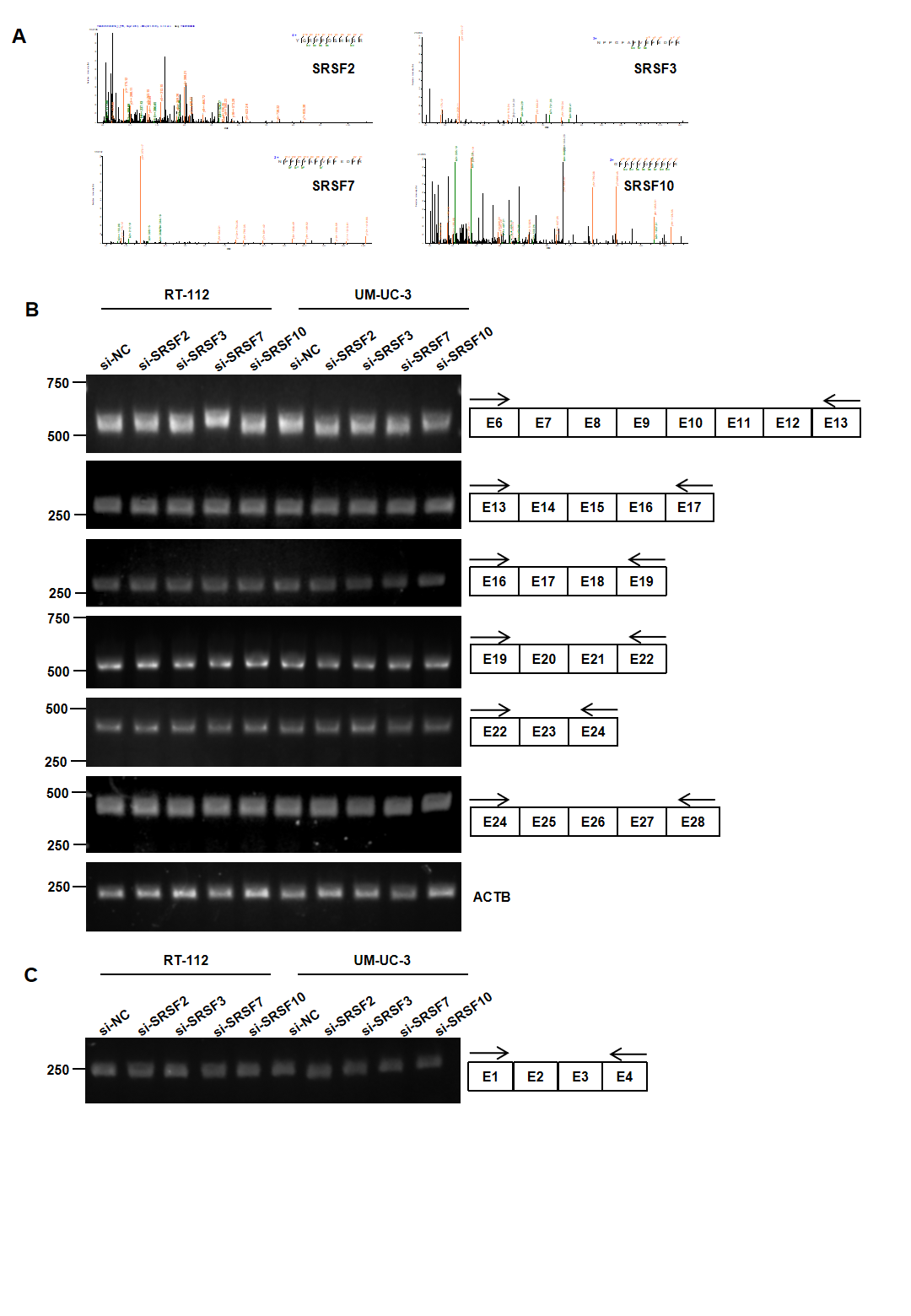


**Supplementary Figure S7. Identification of SR Proteins Interacting with TTK and Their Role in ULK1 mRNA Splicing.**

(A) Peptide maps of SRSF2, SRSF3, SRSF7, and SRSF10 pulled down by Flag-TTK. (B and C) Representative gel images from RT-PCR experiments showing ULK1 mRNA splicing products in RT-112 and UM-UC-3 cells transfected with si-NC, si-SRSF2, si-SRSF3, si-SRSF7 or si-SRSF10. Each product was confirmed by DNA sequencing. ACTB was used as an internal control.


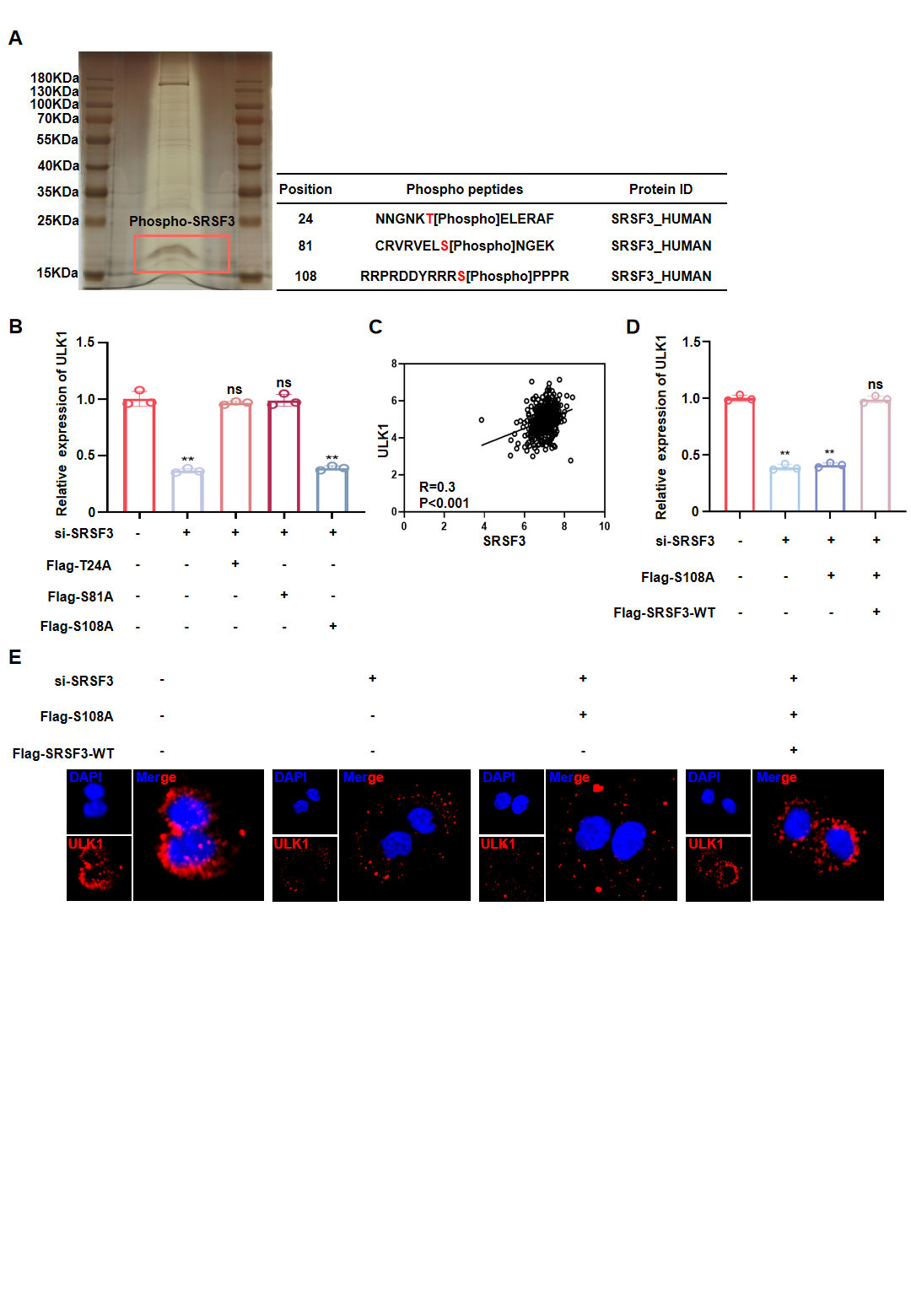


**Supplementary Figure S8. Phosphorylation of SRSF3 by TTK and Its Impact on ULK1 Expression.**

(A) Silver staining showing phosphorylated SRSF3 protein from the in vitro kinase assay, with phosphorylation sites identified by mass spectrometry. (B) qRT-PCR detection of ULK1 mRNA expression in RT-112 cells with SRSF3 phosphorylation site mutations. (C) Pearson correlation analysis indicates a significant positive correlation between the expression of ULK1 and SRSF3 in the TCGA BLCA dataset. (D) qRT-PCR determination of ULK1 mRNA expression in RT-112 cells with SRSF3 phosphorylation site mutations, followed by transfection with plasmid encoding Flag-tagged wild-type SRSF3 (Flag-SRSF3-WT). (E) Confocal microscopy to detect ULK1 in RT-112 cells with SRSF3 phosphorylation site mutations, followed by transfection with plasmid encoding Flag-tagged wild-type SRSF3 (Flag-SRSF3-WT). (Scale bar: 10 μm). Data are presented as mean ± SD from three independent experiments. ns, no significance; ***P* < 0.01.
