# Supplementary material for: TTK promotes mitophagy by regulating ULK1 phosphorylation and pre-mRNA splicing to inhibit mitochondrial apoptosis in bladder cancer
Source: Cell Death Differ. 2025 Apr 23;32(9):1691–706. doi: 10.1038/s41418-025-01492-w (PMC12432130; doi:10.1038/s41418-025-01492-w)

Full and uncropped western blots.

Figure 1C

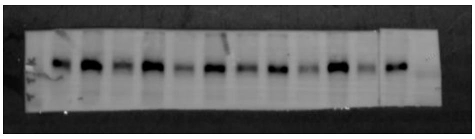

TTK (case1-6)

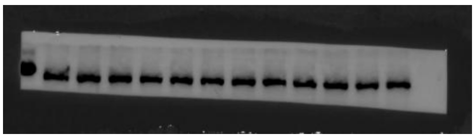

β-actin (case1-6)

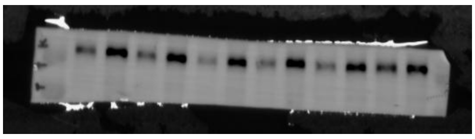

TTK (case7-12)

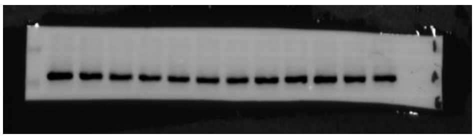

β-actin (case7-12)

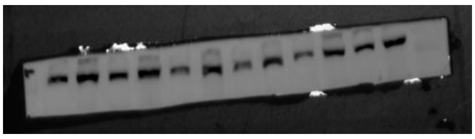

TTK (case13-18)

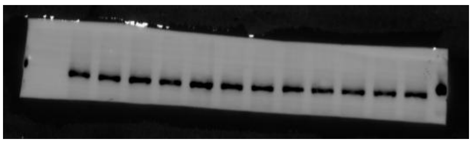

β-actin (case13-18)

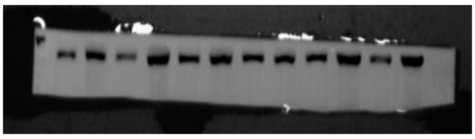

TTK (case19-24)

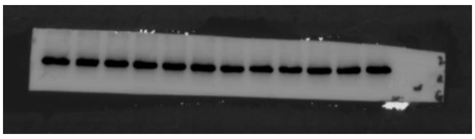

β-actin (case19-24)

Figure 1F

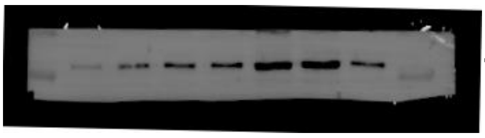

TTK

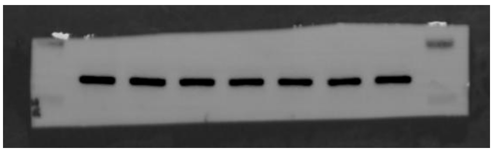

β-actin

Figure 2B RT-112

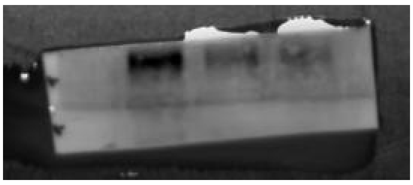

TTK

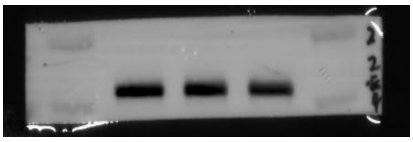

β-actin

Figure 2B UM-UC-3

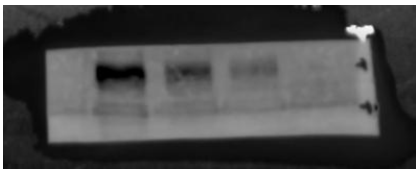

TTK

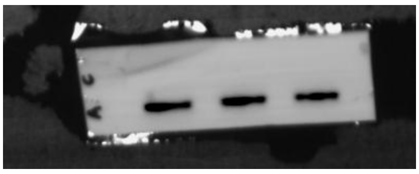

β-actin

Figure 3F RT-112

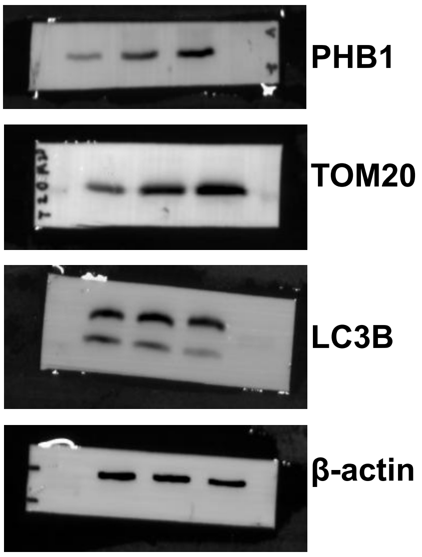

Figure 3F UM-UC-3

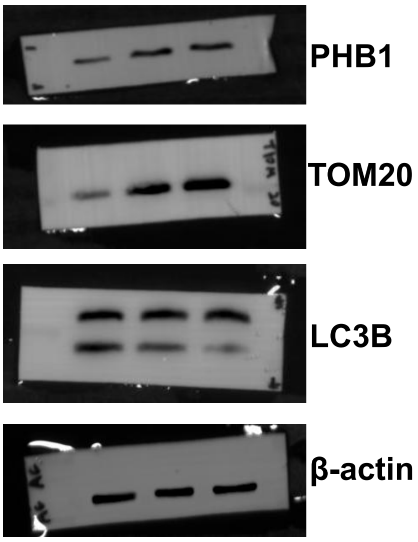

Figure 3G RT-112

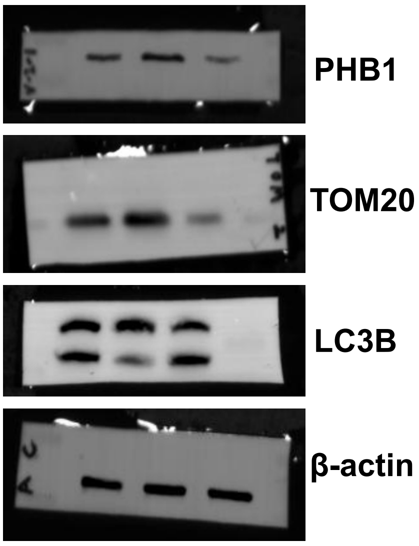

Figure 3G UM-UC-3

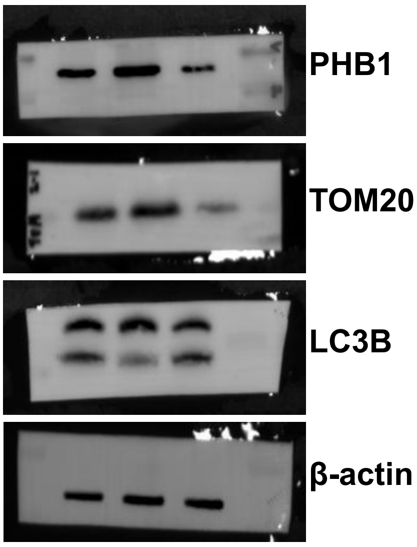

Figure 4F RT-112

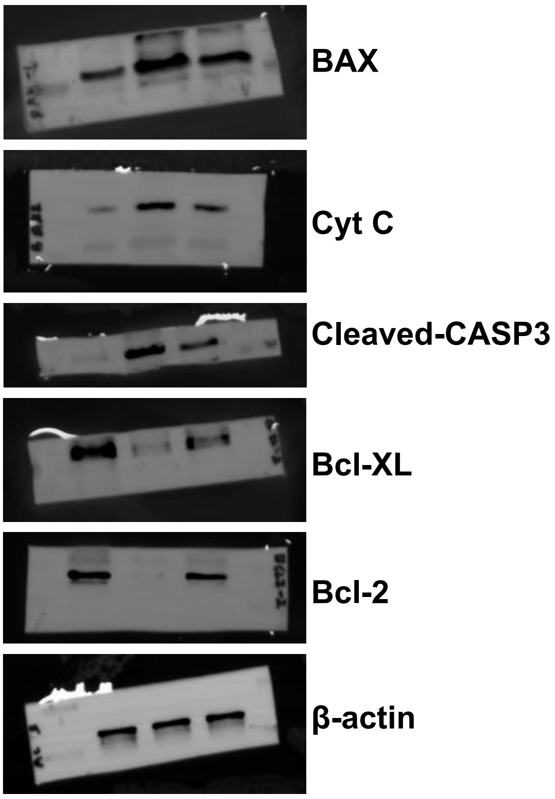

Figure 4F UM-UC3

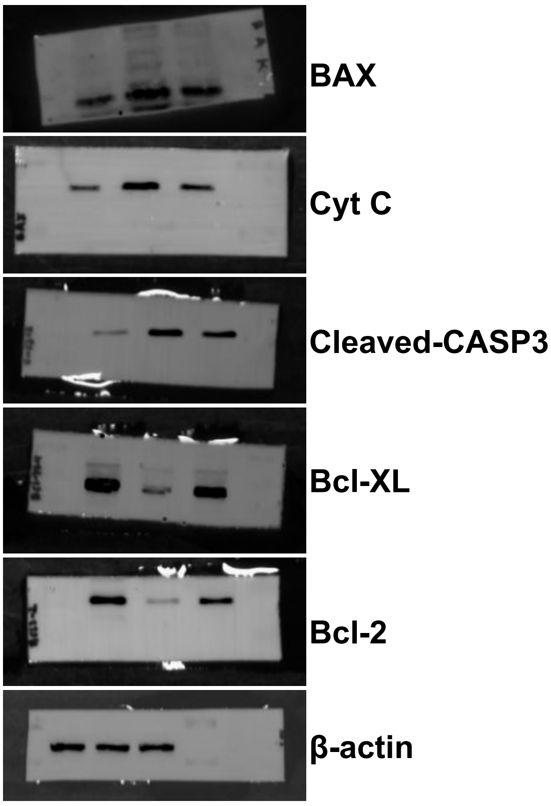

Figure 5D RT-112

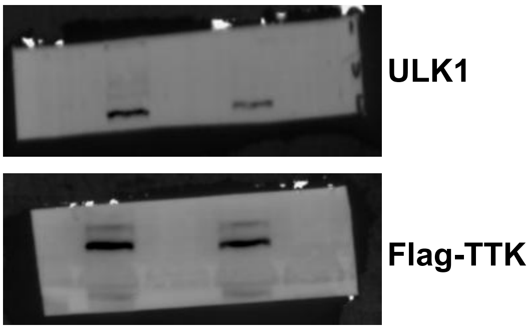

Figure 5D UM-UC-3

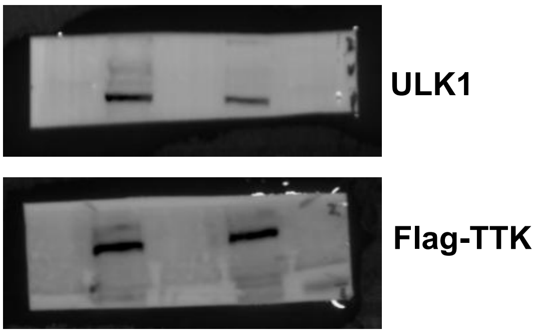

Figure 5F

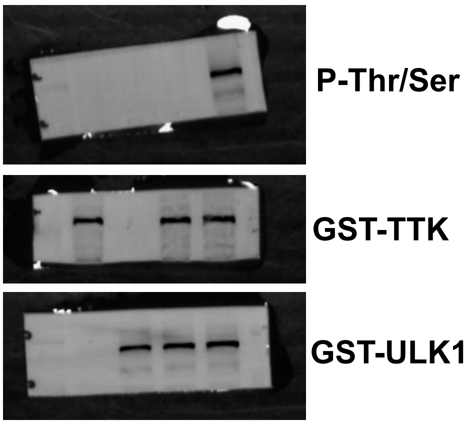

Figure 5I

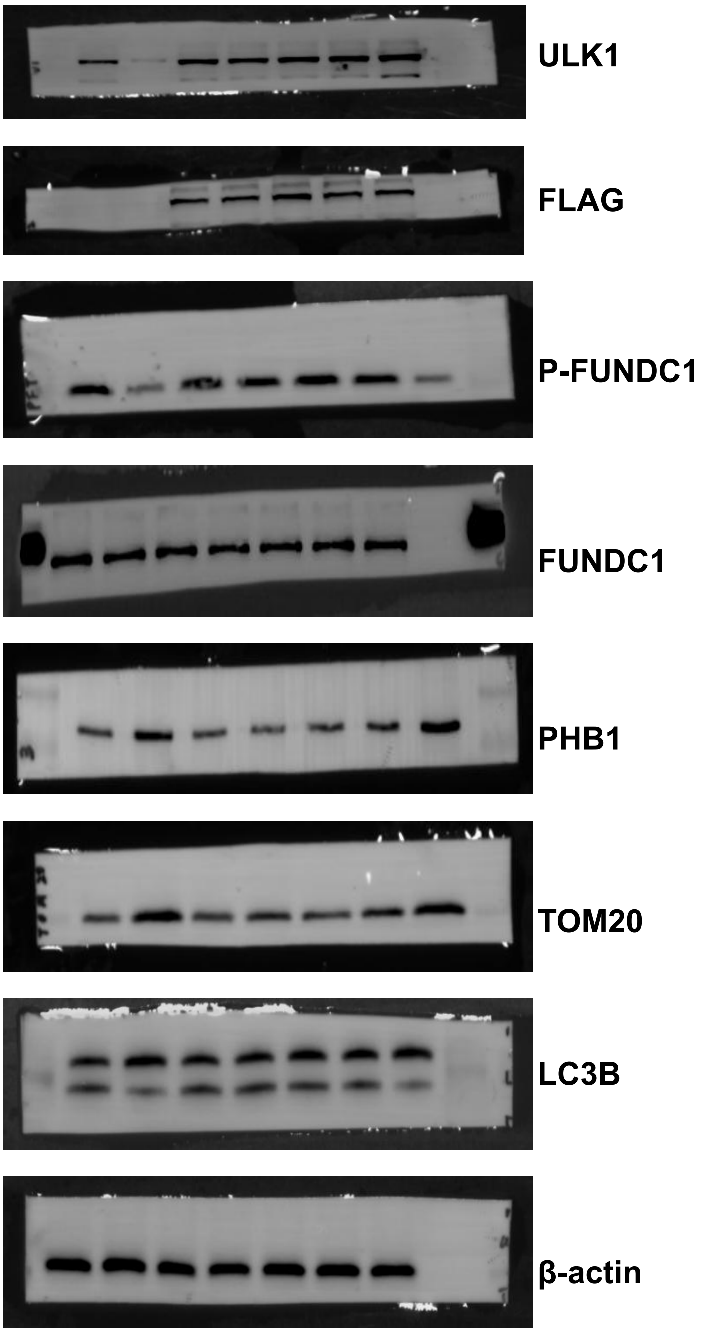

Figure 5J

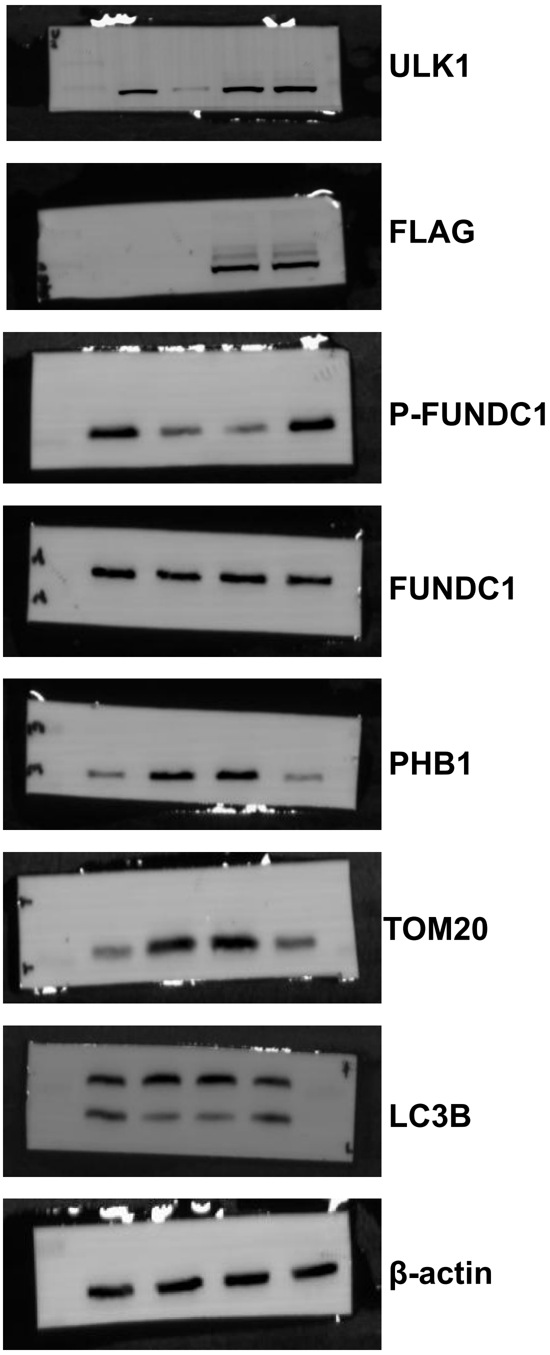

Figure 5K

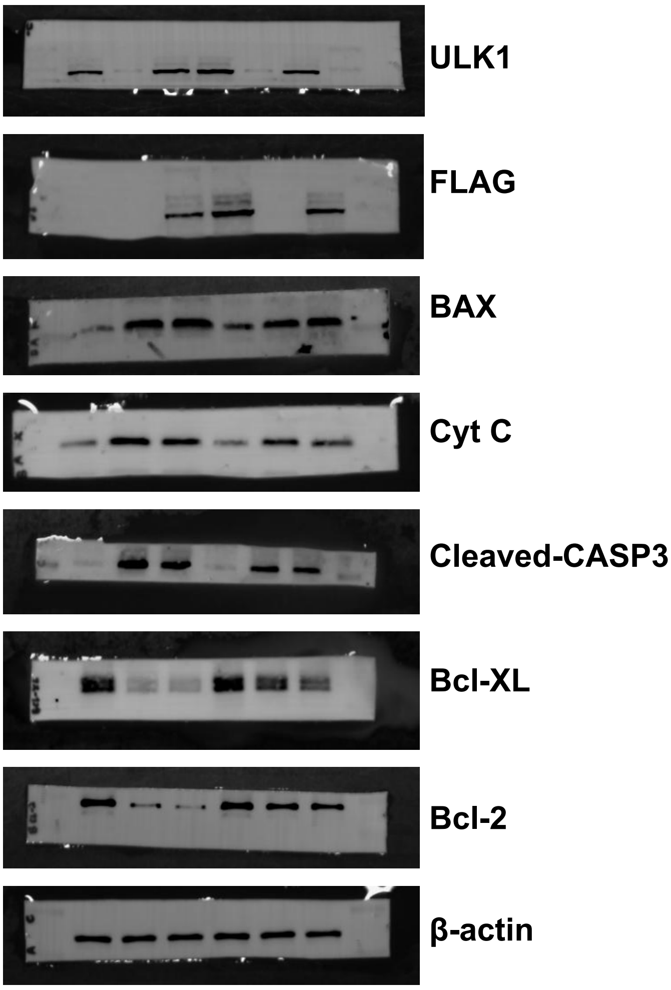

Figure 6A RT-112

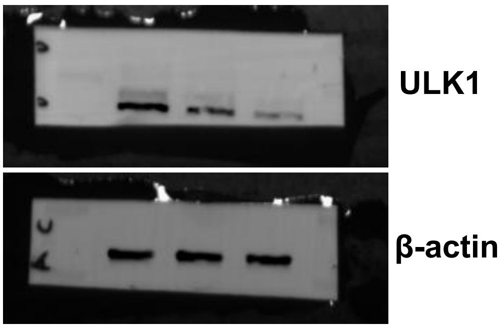

Figure 6A UM-UC-3

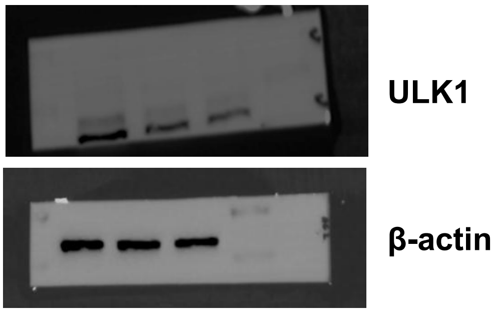

Figure 7B

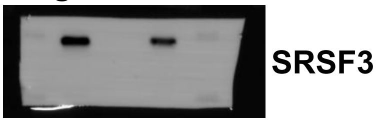

Figure 7E RT-112

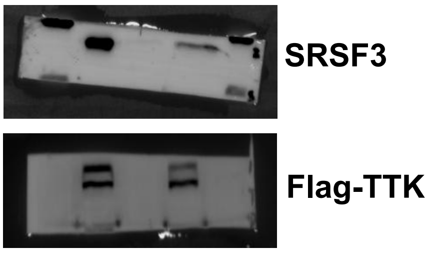

Figure 7E UM-UC-3

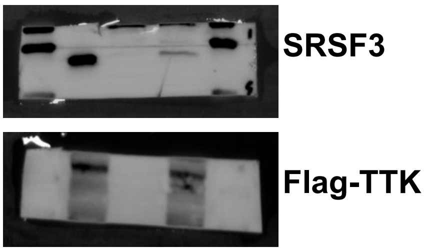

Figure 7F

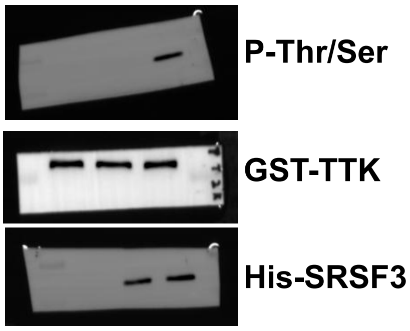

Figure 7H

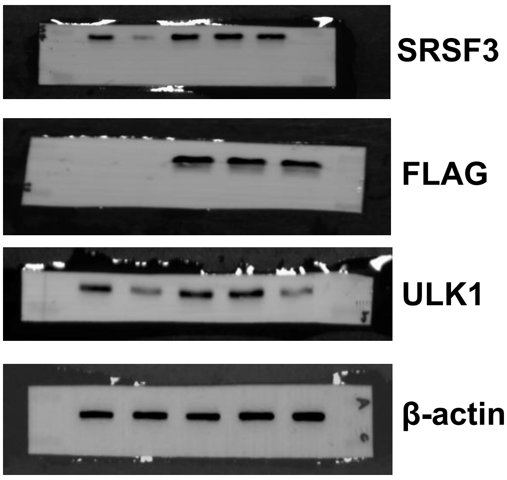

Figure 7J

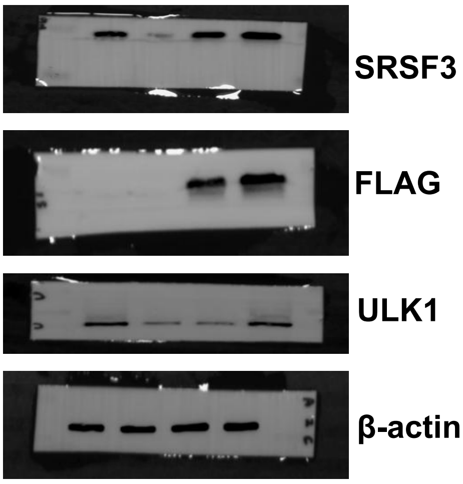

Figure S6A

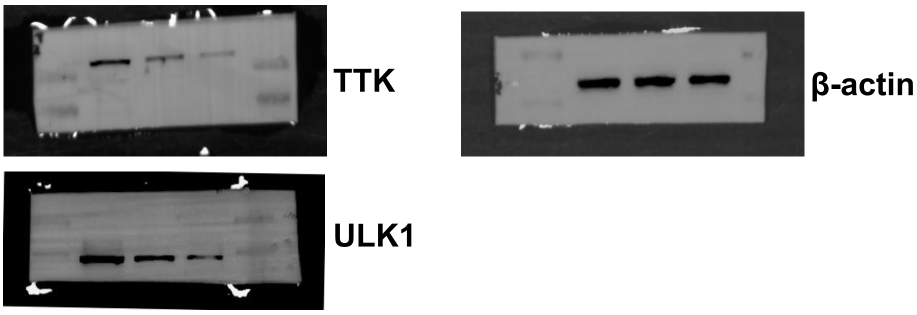

Full and uncropped gel images.

FIGURE 6D

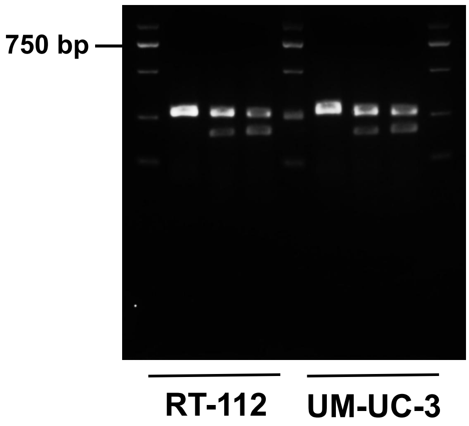

FIGURE 6F

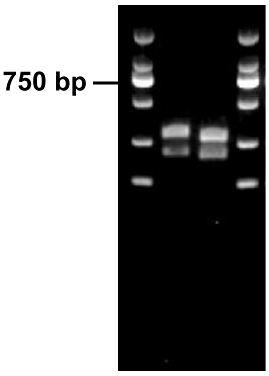

FIGURE S6E

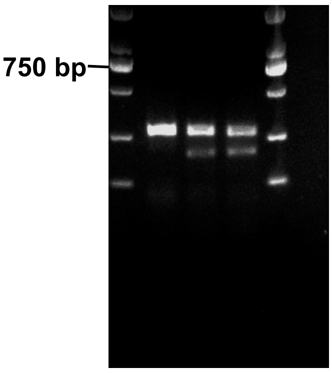

FIGURE 7A RT-112

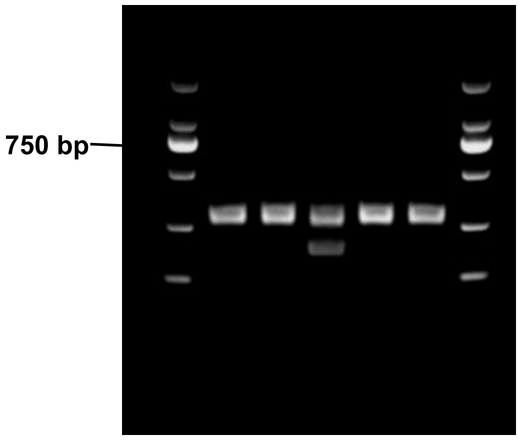

FIGURE 7A UM-UC-3

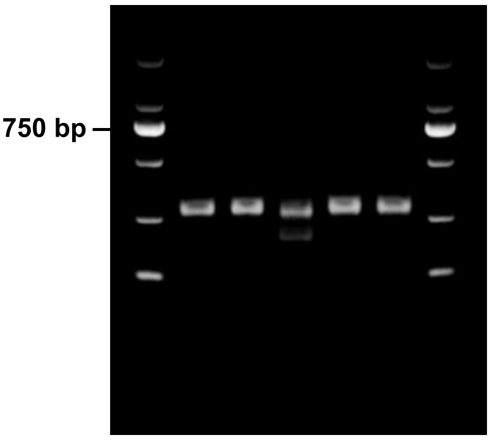

FIGURE 7D

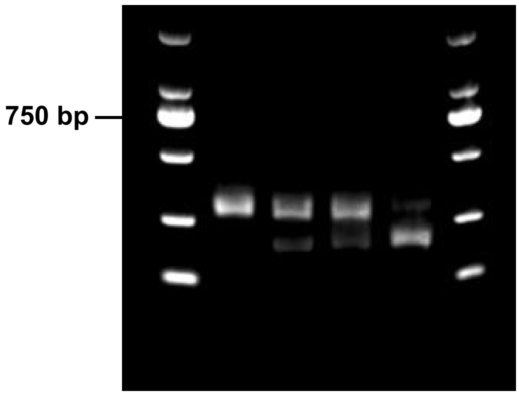

FIGURE 7G

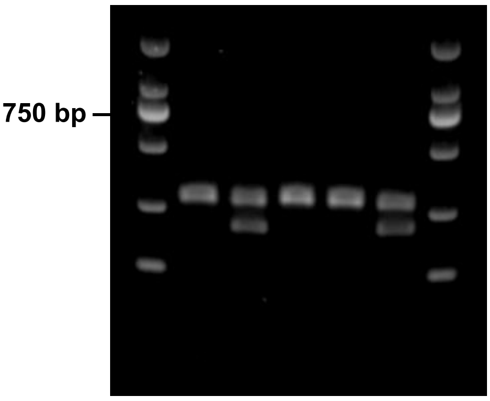

FIGURE 7I

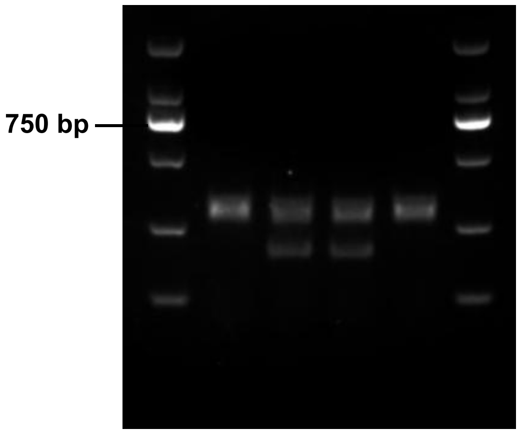

FIGURE S6F

FIGURE S6F

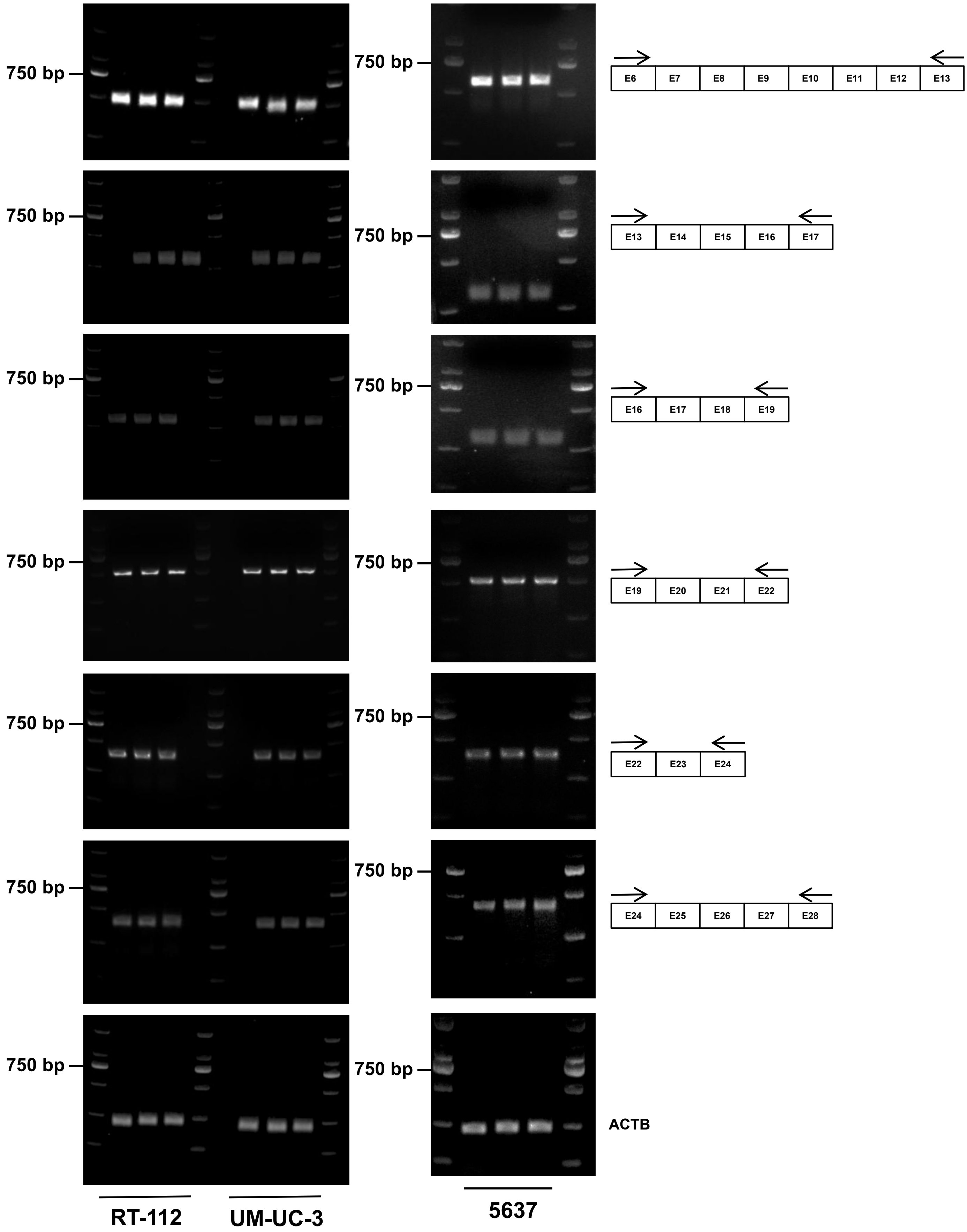

FIGURE S6G

FIGURE S6G

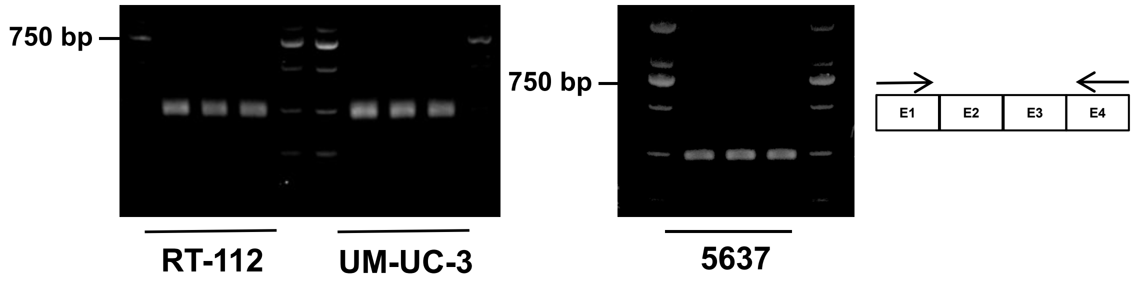

FIGURE S7B

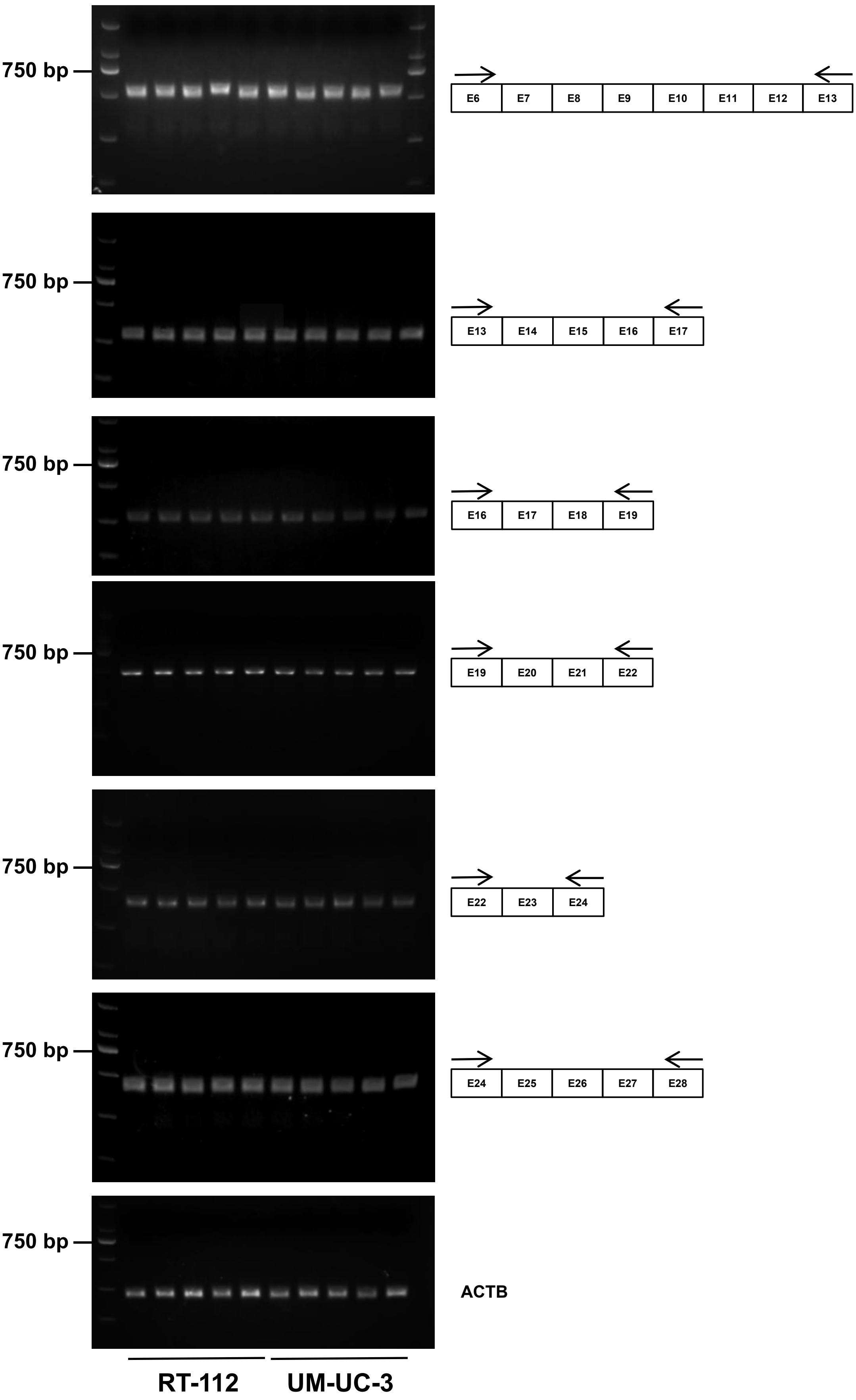

FIGURE S7C

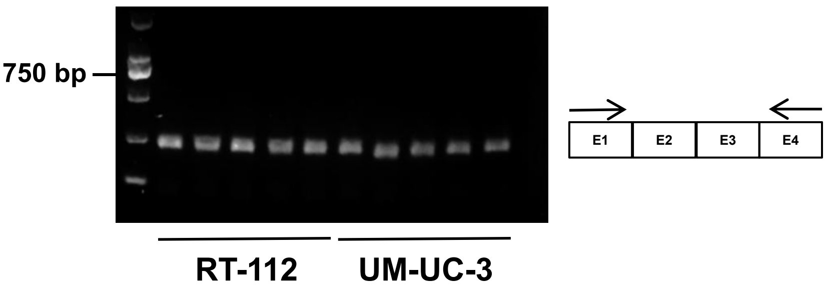

Supplement: Supplementary file 3 — Uncropped blot and gel images [file 41418_2025_1492_MOESM3_ESM.pdf]
